# Copper homeostasis and copper-induced cell death in tumor immunity: implications for therapeutic strategies in cancer immunotherapy

**DOI:** 10.1186/s40364-024-00677-8

**Published:** 2024-10-31

**Authors:** Suhang Zhang, Qibo Huang, Tuo Ji, Qilin Li, Chuanyu Hu

**Affiliations:** 1 Department of Stomatology, Tongji Hospital, Tongji Medical College, Huazhong University of Science and Technology, Wuhan, 430030 China; 2 School of Stomatology, Tongji Medical College, Huazhong University of Science and Technology, Wuhan, 430030 China; 3 Hubei Province Key Laboratory of Oral and Maxillofacial Development and Regeneration, Wuhan, 430030 China; 4 Hepatic Surgery Center, Tongji Hospital, Tongji Medical College, Huazhong University of Science and Technology, Wuhan, 430030 China; 5 School of Medicine, New York Medical College, Valhalla, NY 10595 USA

**Keywords:** Copper homeostasis, Copper metabolism, Cuproptosis, Cancer immunotherapy

## Abstract

Copper is an important trace element for maintaining key biological functions such as cellular respiration, nerve conduction, and antioxidant defense. Maintaining copper homeostasis is critical for human health, and its imbalance has been linked to various diseases, especially cancer. Cuproptosis, a novel mechanism of copper-induced cell death, provides new therapeutic opportunities for metal ion regulation to interact with cell fate. This review provides insights into the complex mechanisms of copper metabolism, the molecular basis of cuproptosis, and its association with cancer development. We assess the role of cuproptosis-related genes (CRGs) associated with tumorigenesis, their importance as prognostic indicators and therapeutic targets, and the impact of copper homeostasis on the tumor microenvironment (TME) and immune response. Ultimately, this review highlights the complex interplay between copper, cuproptosis, and cancer immunotherapy.

## Introduction

Copper, an essential micronutrient, is intricately involved in a plethora of biological processes, including but not limited to, cellular respiration, neurotransmission, and antioxidant defense [1, 2]. Its quintessential role as a cofactor for a variety of enzymes underscores the delicate balance required for copper homeostasis within the human body. However, the perturbation of this balance has been implicated in a spectrum of pathological conditions, with cancer being a notable example where copper dysregulation is observed [3]. The discovery of cuproptosis, a copper-mediated form of regulated cell death, has opened new avenues in understanding the complex interplay between metal ion homeostasis and cell fate [4]. Cuproptosis presents a unique mechanism that is tightly regulated and can be harnessed for therapeutic purposes. The delineation of this novel cell death pathway has not only advanced our comprehension of copper’s role in cellular physiology but also highlighted its potential as a target for cancer therapy [5].

While prior studies have shed light on the involvement of copper metabolism and deposition in tumorigenesis, the specific functions and mechanisms of copper metabolism within the TME, particularly in the context of tumor therapy and immunotherapy, remain unclear. The present review aims to consolidate recent research findings, explore the molecular mechanisms governing copper metabolism and deposition in cancer, evaluate the prognostic significance of CRGs in cancer patients, and investigate their role in influencing the immune response by affecting the TME, ultimately suggesting a new approach for cancer immunotherapy.

### Copper metabolism

**Fig. 1:**
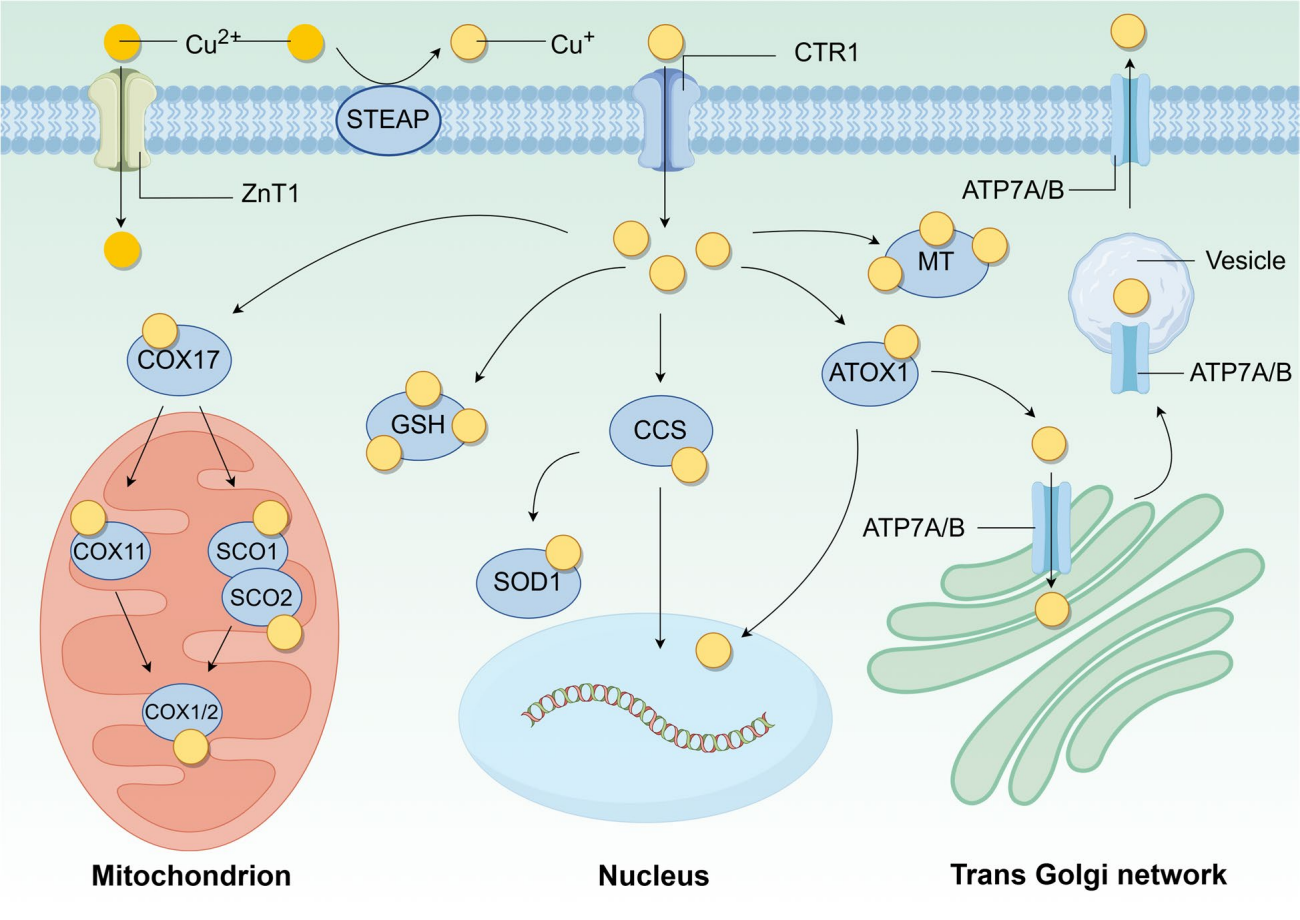
The mechanism of copper metabolism. The complex metabolic mechanism of copper helps to maintain its homeostasis. Cu^2+^ can be transported by ZnT1, or Cu^2+^ is reduced to Cu^+^ by STEAP on the cell membrane and then transported into the cell by SLC31A1/CTR1. After entering the cell, copper is bound by different copper-binding proteins such as COX17, CCS, and ATOX1 for transportation to different organelles such as mitochondria and nucleus to play its role, and the excess free copper is stored by GSH and MT binding to protect the cell. Copper efflux is handled by ATP7A/B, which is localized on the trans Golgi network

Copper metabolism is mainly accomplished through absorption, utilization, and excretion. Here, the mechanisms of copper metabolism in the human body will be summarized (Fig. 1).

#### Copper uptake

The human body primarily obtains copper from the diet such as shellfish, nuts, and animal offal, which are a significant source of this essential mineral. The recommended daily copper intake for adults is 0.8–2.4 mg, with an average absorption rate of copper from food estimated to be 50% [6]. Copper is absorbed in the small intestine and transported to the portal vein by the ATPase copper transporting alpha (ATP7A), and then transferred to the liver through plasma proteins, albumin, and transferrin [7]. The liver serves as the primary organ responsible for the storage, distribution, and excretion of copper. Subsequently, copper enters the systemic blood circulation and is distributed to all tissues and cells. The absorption of copper is an intricate physiological process that facilitates the safe and effective uptake of this vital trace element from the diet and its subsequent delivery to the various cells and tissues in need. Copper in food predominantly exists in an inorganic form as copper salts, while within living organisms it is found as copper ions in both the reduced state (Cu^+^) and the oxidized state (Cu^2+^).

Here we describe the molecular mechanisms of copper uptake and the associated copper transporter proteins. Firstly, in the presence of gastric acid, the copper salts in food begin to dissolve in preparation for subsequent reduction and absorption. Subsequently, copper absorption is achieved in the small intestine, where small intestinal cells are unable to absorb Cu^2+^ directly. Instead, they must be catalyzed by metal reductases on their surface, such as the six-transmembrane epithelial antigen of prostate (STEAP) family proteins and duodenal cytochrome b (DCYTB), which reduce the Cu^2+^ to Cu^+^ [8]. For Cu^+^ to be absorbed, it must first be transported across the cell membrane via the SLC31 copper-permeable family of transport proteins. These proteins, solute carrier family 31 member 1 (SLC31A1)/copper transporter 1 (CTR1) and solute carrier family 31 member 2 (SLC31A2)/copper transporter 2 (CTR2), facilitate the absorption of Cu^+^ into small intestinal cells and other tissues.

A variety of mechanisms can affect the activity of SLC31A1, thereby regulating copper absorption. Zinc finger protein 711 (ZNF711) recruit histone demethylase JHDM2A to the SLC31A1 promoter, which in turn activates SLC31A1 transcription by decreasing the level of H3K9me2 [9]. In contrast, specificity protein 1 (SP1) acts as a sensor for copper and transcriptionally regulates SLC31A1 expression, the expression of SLC31A1 in turn controls the level of cellular copper [10]. Polypyrimidine tract binding protein 1 (PTBP1) binds directly to SLC31A1 mRNA, resulting in the downregulation of SLC31A1 expression [11]. This is achieved by impairing mRNA stability. In the copper homeostatic system, the functions of SLC31A1 and SLC31A2 are interdependent, with SLC31A1 being essential for maintaining the stability of SLC31A2 [12]. Glucose restriction upregulated AMPK (AMP-activated protein kinase) through reactive oxygen species (ROS) to induce SLC31A1 expression [13]. SLC31A1 expression is regulated by intracellular copper levels, with downregulation of SLC31A1 expression in the presence of copper excess and upregulation of SLC31A1 expression in the presence of copper deficiency. This is a negative feedback regulatory mechanism. Solute carrier family 11 member 2 (SLC11A2), also known as divalent metal transporter protein 1 (DMT1) [14], has been reported to transport copper, which may be a compensatory mechanism in the event of hereditary SLC31A1 deficiency.

In addition to the classical copper transporter protein SLC31A1, recently Li et al. found that zinc transporter protein 1 (ZnT1), a zinc efflux protein, also mediates Cu^2+^ entry into cells. Specifically, structural analysis and functional characterization of ZnT1 showed that Zn^2+^ competes with Cu^2+^ for binding to the major binding site on ZnT1 and that a unique inter-subunit disulfide bond on ZnT1 facilitates Cu^2+^ transport, in addition to which, specific knockdown of the ZnT1 gene in the intestinal epithelium leads to loss of Lgr5^+^ stem cells due to copper deficiency [15].

SLC31A1, a regulatory gene for cuproptosis, has been reported in pan-cancer studies to exhibit higher expression in most tumor types than in non-tumor tissues [16]. SLC31A1 expression is increased in cervical cancer, endometrial cancer (EC) and breast cancer (BC), but decreased in clear cell renal cell carcinoma (ccRCC), hepatocellular carcinoma (HCC) and lung adenocarcinoma (LUAD). The expression of SLC31A1 was positively correlated with immune infiltration, and the single-cell sequencing results indicated that SLC31A1 might be involved in DNA repair, DNA damage and cell proliferation processes in tumor cells [17]. In individuals diagnosed with adrenocortical carcinoma (ACC), low-grade glioma, or mesothelioma (MESO), heightened expression levels of SLC31A1 were correlated with diminished overall and disease-free survival rates. These findings indicate the potential utility of SLC31A1 as a pivotal biomarker and target for therapeutic interventions in various tumor types [18].

#### Copper utilization

Upon entering the cell, copper binds to copper chaperone proteins to form complexes, which are subsequently transported to various cellular compartments, including the cytoplasm, mitochondria, Golgi apparatus, and nucleus. These complexes regulate various cellular activities. Three primary intracellular copper chaperone proteins have been characterized as follows: copper chaperone protein for superoxide dismutase (CCS), antioxidant 1 copper chaperone protein (ATOX1), and cytochrome c oxidase copper chaperone protein 17 (COX17). These proteins will be further delineated for enhanced comprehension.

In the cytoplasm and mitochondria, CCS transports copper to superoxide dismutase 1 (SOD1), which requires copper in its cofactor [19]. CCS and SOD1 are structurally related and co-localized in the cytoplasmic and mitochondrial membrane interstitials. CCS can preferentially bind to the immature form of SOD1, thereby promoting its maturation [20]. In addition, SOD1 protects cells from oxidative stress by catalyzing the conversion of superoxide radicals to oxygen and hydrogen peroxide [21]. Mitochondria produce adenosine triphosphate (ATP) via oxidative phosphorylation, which is an important copper reservoir and a major copper user for intracellular copper-based enzymes [22]. CCS acts as a specific input receptor within mitochondria, facilitating the input and folding of both SOD1 and CCS, thus expanding the spectrum of substrates for the input of oxidative-based proteins into the mitochondrial membrane compartment [23]. The expression of SOD1 is also important in cancer. In addition, SOD1 is overexpressed in BC, and SOD1 is required for oncogene-driven proliferation but not for normal tissue proliferation. SOD1 maintains ROS levels below a threshold and promotes tumorigenesis. SOD1 may also serve as a novel target for cancer therapy [24]. In specific circumstances, copper-induced oxidative stress can be reduced, and/or ribosome biogenesis can be enhanced to promote tumorigenesis by elevated levels of SOD1, as demonstrated in mouse models of HER2-positive BC and KrasG12D-mutant non-small cell lung cancer (NSCLC) [25]. CCS also interacts directly with and transfers copper to the copper enzymes mitogen-activated extracellular signal-regulated kinase 1 and 2 (MEK1 and MEK2), thereby playing a crucial role in activating their kinase activities. MEK1 and MEK2 are components of downstream oncogenic RAS and RAF signaling pathways and are important mediators of malignant transformation and drug resistance [26].

Copper is transported to the trans-Golgi network via ATOX1. In the cytoplasm, ATOX1 binds Cu^+^, which is subsequently delivered to the ATP7A and ATPase copper transporting beta (ATP7B) in the Golgi network [27]. In the presence of elevated copper levels, ATP7A/B are relocated to post-Golgi sites, such as lysosomes or melanosomes, where they facilitate the translocation of copper from the Golgi to post-Golgi sites, thereby promoting the efflux of excess copper [28, 29]. The mediator of ERBB2-driven cell motility 1 (MEMO1), a protein associated with HER2-driven cell motility and migration, can form a ligand complex with Cu^+^ and translocate it to ATOX1, thereby inhibiting copper-mediated redox activity and ROS production [30]. ATOX1 has been associated with tumorigenesis [31]. It has been shown that increased ATOX1 expression leads to resistance to genotoxic drugs in a variety of cancer cells [32]. In BC, ATOX1 enhances the migration of BC cells by activating the copper-dependent protein lysyl oxidase (LOX) [33, 34]. DC_AC50 is a selective small molecule inhibitor against ATOX1 that blocks the binding of ATOX1 to copper, thereby increasing intracellular copper accumulation. By inhibiting ATOX1, DC_AC50 enhances the chemotherapeutic effect of platinum-based drugs such as cisplatin [35]. Additionally, it is observed that cisplatin can bind to ATOX1 regardless of the presence of copper. Subsequently, as time progresses, the stability of this interaction diminishes, leading to protein unfolding and aggregation. This suggests that ATOX1 could serve as a promising target in combating cisplatin resistance, potentially hindering the delivery of cisplatin to DNA by binding to ATOX1 within the cytoplasm [36]. Both CCS and ATOX1 transport copper to the nucleus, where copper upregulates hypoxia-inducible factor 1 (HIF1) activity [37]. These activities of ATOX1 are dependent on its highly conserved C-terminal KKTGK motif and N-terminal copper-binding site [38]. Inhibition of CCS and/or ATOX1 results in copper mislocalization and/or impaired function of copper-dependent effector proteins, thereby inhibiting cuproplasia and tumor growth in various in vitro and in vivo models [31, 39, 40].

Copper is a vital cofactor in the production of energy through mitochondrial respiration [22]. COX17 facilitates the transfer of Cu^+^ from the cytoplasm into the inner mitochondrial membrane, subsequently delivering copper to the synthesis of cytochrome c oxidase 1 (SCO1), where they form disulfide bonds [41]. Cytochrome c oxidase assembly factor 6 (COA6), a thiol-disulfide oxidoreductase, reduces the formation of disulfide bonds between cysteine residues in the synthesis of SCO1/2 and substances other than copper, thereby allowing copper binding. Conversely, the absence of COA6 results in impaired respiratory complex IV biosynthesis. In the presence of COA6 and COX16, SCO1 and SCO2 transfer copper obtained from COX17 to mitochondria-encoded cytochrome c oxidase subunit 2 (MT-CO2; aka COX2) [42, 43]. Another pathway by which COX17 transports copper from the cytoplasm to mitochondria-encoded cytochrome c oxidase 1 (MT-CO1/COX1) is through the transfer of copper to cytochrome c oxidase copper chaperonin 11 (COX11) [44]. The activity of complex IV in the respiratory chain reaction is largely dependent on its cofactor copper in the mitochondria [45]. Slight changes in copper levels may disrupt the mitochondria. MT-CO1/MT-CO2 is the copper-binding subunit of complex IV that transfers electrons from cytochrome c somatic cells (CYCS) and initiates the electrochemical production of ATP. Furthermore, SCO1 and SCO2 are involved in the regulation of cellular copper homeostasis. The absence of both has been observed to result in decreased cellular copper levels [46]. The maintenance of intracellular copper homeostasis is contingent upon the interaction of these four proteins (COX17, COX11, SCO1, and SCO2). Additionally, COA6 has been identified as a potential biomarker and therapeutic target for cancer, with a strong association with tumor prognosis and immunotherapy outcomes [47]. For instance, high expression of COA6 is linked to increased oxidative phosphorylation and a poor prognosis in LUAD patients [48].

Inside the cell, copper is bound to copper chaperone proteins, but it is also can be free. Free copper can damage cells by generating ROS or exerting cytotoxic effects, while metallothionein (MT) and glutathione (GSH) chelate this excess copper to participate in the regulation of intracellular copper homeostasis [40]. MT is a class of cysteine-rich small proteins containing heavy metal-bound MT clusters [49], MT1 and MT2 can chelate the large amount of copper transported by SLC31A1, and the addition of metal ions such as copper induces MT expression [50]. In the absence of MTs, Cu transport of ATP7A from the trans-Golgi network to cytoplasmic vesicles is stimulated, suggesting that MTs regulate the availability of Cu for ATP7A transport [51]. Although MT expression is not prevalent in all human tumors, increasing evidence suggests that MT plays a critical role in tumor initiation, progression, and drug resistance [52]. GSH is the most abundant non-protein sulfhydryl group, and GSH binds excess free copper before MT-mediated copper binding [53]. In tumor cells, elevated levels of GSH have been found to correlate with tumor progression and increased resistance to chemotherapeutic agents [54].

#### Copper excretion

At the systemic level, the liver serves as the primary storage site for copper, with excess copper predominantly excreted through bile. Alternative pathways for copper excretion, including urine, sweat, and menstruation, have a comparatively minimal effect on overall copper elimination [55]. The regulation of systemic copper status is primarily governed by the processes of duodenal absorption and biliary excretion. In instances of elevated copper intake, absorption of copper is attenuated, leading to an increase in copper excretion. Conversely, during periods of low copper intake, the excretion of copper through the bile is reduced, while the retention of absorbed copper is increased [56].

At the cellular level, copper efflux depends on the copper efflux transporter proteins ATP7A/B [57]. ATP7A is expressed in most tissues, whereas ATP7B is essential in the liver for the homeostatic regulation of systemic copper levels, and its mutation leads to Menkes’ disease [58] and Wilson’s disease [28], respectively. Under basal conditions of low copper levels, ATP7A and ATP7B are localized in the trans-Golgi network. With increasing copper exposure, ATP7A and ATP7B translocate to cell membranes or intracellular vesicles, and then ATP7A and ATP7B translocate copper from the trans-Golgi network to post-Golgi vesicles. These copper-carrying vesicles can fuse with the plasma membrane and release copper into the extracellular environment [59]. This process requires energy from ATP to transport copper against its concentration gradient [60]. Moreover, the activities of ATP7A and ATP7B are tightly regulated by intracellular copper levels and copper-binding proteins (e.g. MT) [51], as well as by various signaling pathways that regulate the transport and activity of these transporter proteins.

The abundance of ATP7A in a variety of tumor cell lines was found to correlate with increased resistance to cisplatin, a widely used chemotherapeutic agent, and tumor transplants deficient in ATP7A were significantly more sensitive to cisplatin chemotherapy than control tumors expressing ATP7A. Deletion of ATP7A significantly inhibited tumorigenesis in H-RAS transformed mouse embryonic fibroblasts (MEF^RAS^7A-) [61]. This phenomenon was linked to copper hyperaccumulation and susceptibility to ROS and hypoxia. Furthermore, the upregulation of ATP7A and ATP7B expression is implicated in conferring resistance to platinum-based chemotherapeutic agents in human ovarian cancer (OC) cells [60]. These studies demonstrate the potential of ATP7A/B as a potential therapeutic target for the regulation of tumor growth and the efficacy of platinum-based therapies [62, 63]. It was found that ATP7A was generally highly expressed in digestive system tumors and was associated with poor prognosis in HCC [64]. Furthermore, ATP7A expression was found to be positively correlated with the infiltration of immune cells, including CD3^+^ T cells and CD8^+^ T cells, as well as with the expression of immune checkpoints, particularly programmed cell death ligand 1 (PD-L1). Patients with HCC exhibiting concurrent expression of ATP7A and PD-L1 demonstrate a poorer prognosis.

The imbalance of copper homeostasis is highly correlated with the hallmarks of cancer. An elevated copper level is observed in the majority of cancers, which is a remarkable hallmark of cancer [65]. Copper participates in and promotes cancer growth, angiogenesis, invasion, metastasis, etc [66]. One feature of Wilson’s disease is the progressive copper accumulation mainly in the liver, which can lead to severe cellular damage [67], and Wilson’s disease has the risk of developing into HCC [68]. Copper can activate the HIF-1α/GPER/VEGF signaling pathway in cancer cells, facilitating angiogenesis and tumor growth [69]. Additionally, the copper molecular chaperone ATOX1 has been proven to play a role in the migration of BC cells, and migration is a crucial step in cancer invasion and metastasis [34]. Targeting copper homeostasis can effectively treat cancer, and related therapies such as copper ionophores and copper chelators will be elaborated in detail in the following sections.

#### The mechanism of cuproptosis

**Fig. 2:**
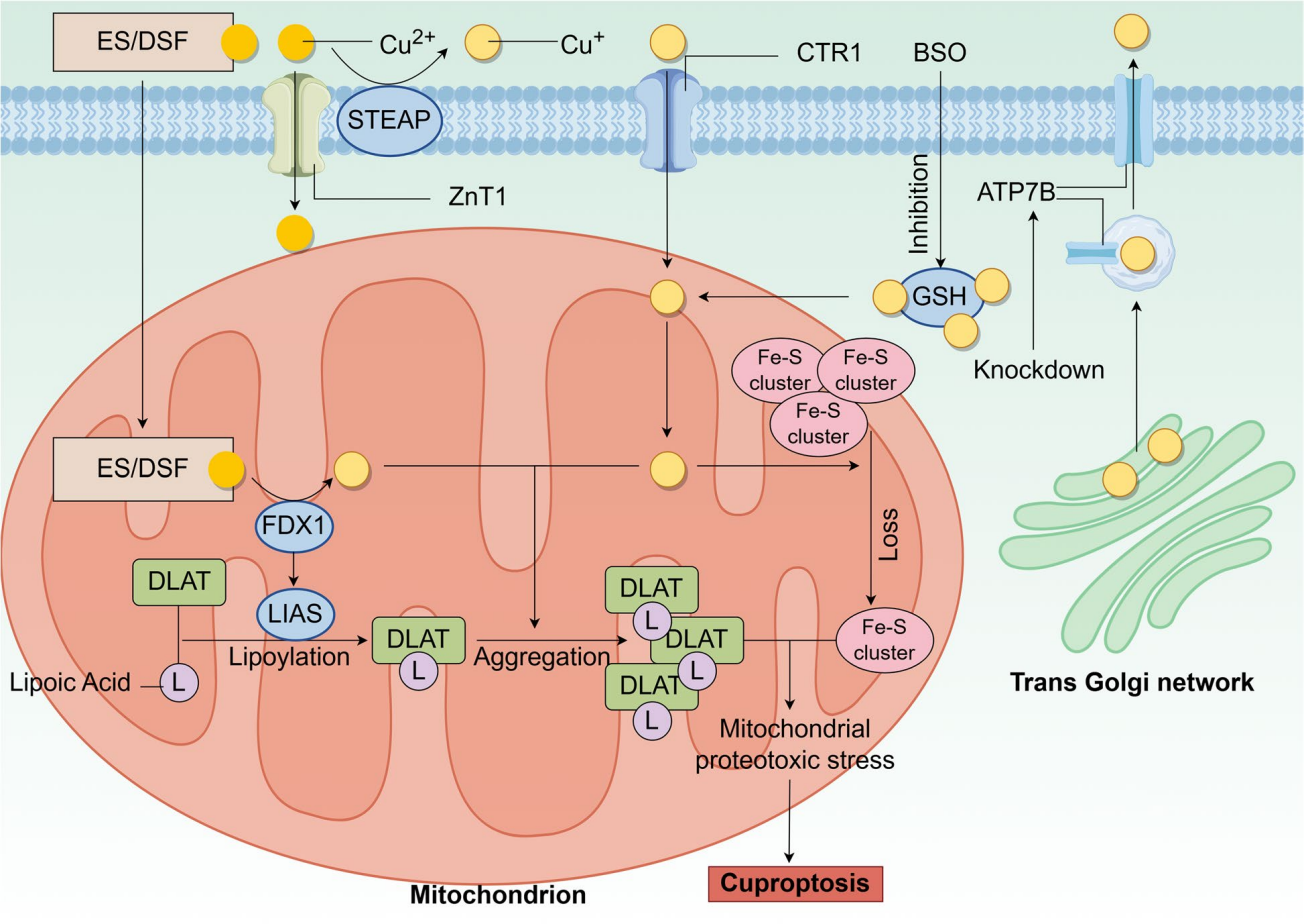
The mechanism of cuproptosis. Cuproptosis is triggered by excessive accumulation of intracellular copper, which can be achieved in several ways: (1) Abnormalities in copper transport proteins, such as increased expression of SLC31A1, ZnT1 or decreased ATP7B. (2) Direct transport of Cu^2+^ into the cell via the copper ion carrier ES or DSF. (3) Inhibition of the synthesis of the copper chelator GSH by BSO leading to copper release from GSH. Excess copper subsequently leads to the reduction of Fe-S clusters, aggregation of lipoylated DLAT, and ROS production, which causes mitochondrial proteotoxic stress leading to cuproptosis

Intracellular copper maintains homeostasis under normal conditions, but in the presence of excess copper leads to copper-induced cell death, known as cuproptosis. Cuproptosis is a novel mode of cell death proposed by Tsvetkov et al. in 2022 [4]. Cuproptosis is a unique cell death mechanism that is distinct from other known cell death mechanisms, including apoptosis [70], autophagy [71], ferroptosis [72], and others. Next, we will briefly introduce the mechanism of cuproptosis (Fig. 2).

Cuproptosis occurs on the premise that there is an imbalance in intracellular copper homeostasis, which means that copper has to accumulate in excess, and this step can be achieved in several ways: (1) Abnormalities in copper transporter proteins such as increased expression of SLC31A1/CTR1 and ZnT1 leading to an increase in copper entering the cell or a decrease in ATP7B leading to a decrease in Cu^+^ efflux [28, 73]. (2) Direct transporter of Cu^2+^ by copper ionophores such as elesclomol (ES) or disulfiram (DSF) into the cell [74, 75]. (3) Inhibition of the synthesis of the copper chelator GSH by buthionine sulfoximine (BSO) leads to the release of free copper from GSH, resulting in increased intracellular copper levels [76]. Subsequently, a complex series of intracellular reactions occur: in the mitochondria [77], ferredoxin 1 (FDX1) reduces Cu^2+^ to the more toxic Cu^+^, a process that generates ROS, Cu^+,^ and ROS promotion leads to a reduction in the synthesis of iron-sulfur (Fe-S) clusters, and FDX1 can bind to lipoic acid synthetase (LIAS) to promote the lipoylation of dihydrolipoamide S-acetyltransferase (DLAT) [78], which serves as an important component of the pyruvate dehydrogenase complex to regulate the mitochondrial tricarboxylic acid (TCA) cycle. Moreover, Cu^+^ promotes further aggregation of lipoylated DLAT.

After the above series of reactions, the reduction of Fe-S clusters, aggregation of lipoylated DLAT, and ROS production combine to cause mitochondrial proteotoxic stress, leading to cuproptosis. Recently, it has been found that tumor suppressor p53 can promote or prevent cuproptosis through a variety of mechanisms [79]. Specifically, firstly, p53 can regulate intracellular levels of copper, thereby maintaining appropriate copper concentrations and preventing copper-induced cytotoxicity [75]. Second, p53 can regulate the synthesis of Fe-S clusters and GSH [80, 81], which are key components of the cuproptosis pathway. In addition, p53 can promote cuproptosis by enhancing mitochondrial metabolism. For cancer cells, which prefer glycolysis (Warburg effect) over oxidative phosphorylation to produce intermediate metabolites and energy, enabling resistance to cuproptosis [82], p53 inhibits glycolysis and drives the metabolic switch to oxidative phosphorylation in cancer cells. This could provide new perspectives on the use of cuproptosis for cancer treatment. However, further studies are needed to gain insight into the relationship between p53 and cuproptosis.

## Cuproptosis in cancers

**Fig. 3:**
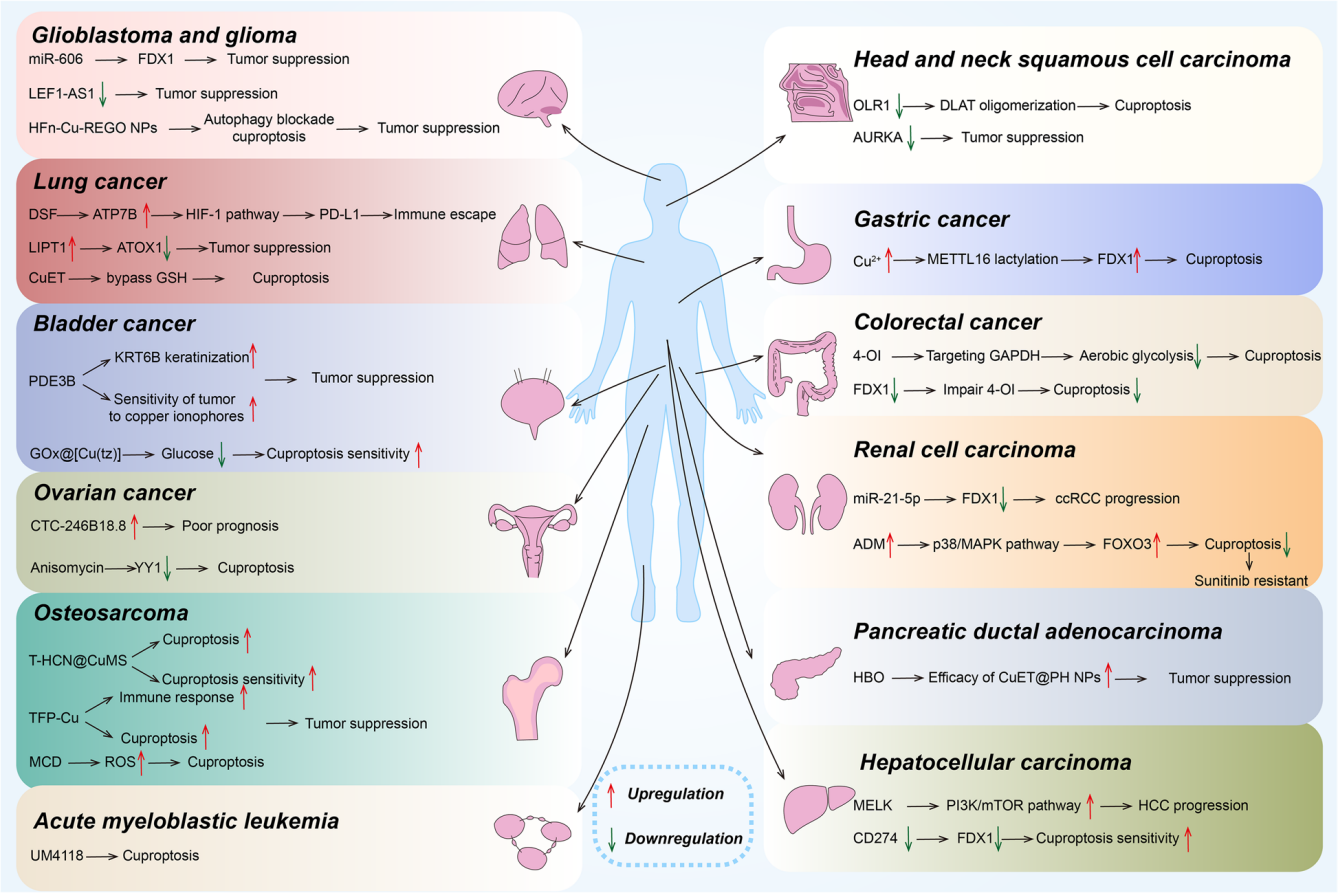
Cuproptosis in cancers

A considerable number of studies have demonstrated that elevated copper levels have been significantly observed in tumor tissues and serum [83]. Furthermore, cuproptosis is found to be significantly linked to tumorigenesis and development, providing a new perspective for tumor treatment [84]. Many researchers have investigated the link between cuproptosis and tumor progression through bioinformatics. CRGs are potentially involved in many cancer types and can be developed as candidates for cancer diagnosis, prognosis, and therapeutic biomarkers [85]. Here, we will introduce seven CRGs that promote cuproptosis and three CRGs that inhibit it, as well as discuss advances in cuproptosis research in different cancers (Tables 1 and 2**and** Fig. 3).

**Table 1:** The role of CRGs and their association with cancers

CRGs	Biological Function	Expression in cancers	Significance of expression		Ref.
FDX1	Transport electrons	Upregulation in COAD, DLBC, GBM, LUAD, OV, PAAD, PRAD, READ, SKCM, STAD, THYM, OS, UCEC, UCS Downregulation in ACC, AML, BRCA, CCA, HNSCC, KICH, ccRCC, KIRP		Low expression → poor prognosis in ccRCC High expression → good prognosis in COAD	[86–93]
DLAT	Participate in the TCA cycle	Upregulation in LIHC, LUAD, OS, LUSC, STAD Downregulation in HNSCC, ccRCC		High expression → poor prognosis in PAAD	[91, 94–96]
LIPT1	Participate in the TCA cycle	Upregulation in HCC, OS, AML, melanoma Downregulation in NSCLC		High expression → poor prognosis in HCC as well as good prognosis in melanoma and NSCLC Low expression → poor prognosis in AML	[89, 91, 97–102]
PDHA1	Participate in the TCA cycle	Upregulation in CSC, CESC, CCA, HCC, LUAD, LUSC, STAD, UCEC Downregulation in BRCA, AML, GBM, ccRCC, KIRP, PCPG, THCA		High expression → poor prognosis in PCA Low expression → poor prognosis in ccRCC	[89, 103–108]
LIAS	Participate in lipoic acid metabolism	Upregulation in CCA, HCC, LUAD, DLBC, THYM, LUSC Downregulation in BRCA, COAD, ccRCC, KIRP, PRAD, READ, THCA, AML, UCEC		High expression → good prognosis ccRCC, READ, BRCA and OC as well as poor prognosis in lung cancer	[78, 97, 109]
PDHB	Participate in the glycolytic pathway	Upregulation in HCC Downregulation in COAD, HNSCC, ccRCC, KIRP, LUSC, READ, THCA		Low expression → good prognosis in ccRCC	[110–113]
DLD	Participate in the TCA cycle	Upregulation in BRCA, CCA, DLBC, GBM, KICH, KIRP, HCC, LUAD, LUSC, PAAD, PRAD, READ, SKCM, STAD, TGCT, THYM Downregulation in ACC, BLCA, ccRCC, AML, PCPG, THCA		High expression → poor prognosis in BLCA and UCS as well as good prognosis in ccRCC and KIRP	[97, 114, 115]
ZnT1	Transport copper and zinc	c in ESCA, PAAD, READ, STAD, THYM Downregulation in HCC		High expression → poor prognosis in ESCA, PAAD, READ, STAD, THYM Low expression → poor prognosis in HCC	[15, 116, 131]
SLC31A1	Transport copper	Upregulation in cervical cancer, EC and BC, Downregulation in ccRCC		High expression → poor prognosis in ACC, BLCA, MESO, SKCM Low expression → poor prognosis in ccRCC	[16–18]
ATP7A	Transport copper	Upregulation in HCC		High expression → poor prognosis in HCC	[57, 64]
CDKN2A	Tumor suppress	Upregulation in ACC, CESC, AML, DLBC, OV, PAAD, PCPG, sarcoma, THYM, UCS Downregulation in TGCT		High expression → poor prognosis in ACC, COAD, ccRCC, HCC, PRAD, SKCM, THCA and UCEC as well as good prognosis in GBM	[118–120, 122, 123]
GLS	Participate in amino acid and nitrogen metabolism	Upregulation in GBM, BC (luminal), lung cancer, PDAC, AML Downregulation in BC (basal)		High expression → tumor growth and proliferation in HCC	[89, 122, 126]
MTF1	Regulate metal ion metabolism and homeostasis	Upregulation in OC, AML Downregulation in GC		High expression → poor prognosis in OC, AML Low expression → good prognosis in GC	[89, 127, 129, 130]

**Table 2:** Cuproptosis and CRGs in cancers

Cancer types	Important CRGs	Advances of cuproptosis in cancer	References
HCC	LIPT1, LIAS, PDHB, DLD, CDKN2A, GLS, DLAT	MELK → PI3K/mTOR pathway→ DLAT↑ → tumor progression	[100, 101, 106, 109, 111, 115, 122, 132–134]
HNSCC	FDX1, DLAT, PDHB, AURKA	OLR1↓ → DLAT oligomerization↑ → cuproptosis AURKA↓ → tumor suppression	[90, 95, 111, 137, 138]
GC	MTF1, SERPINE1, FDX1	Cu↑ → METTL16 lactylation→ FDX1↑ → cuproptosis	[130, 140, 141]
CRC	CDKN2A, DLAT, DLD, PDHB, FDX1	4-OI → targeting GAPDH → aerobic glycolysis↓→ cuproptosis FDX1↓ → impair 4-OI → cuproptosis↓	[142–145]
ccRCC	FDX1, PDHA1, LIAS, PDHB, DLD, CDKN2A	MiR-21-5p → FDX1↓ →tumor progression ADM↑ → p38/MAPK pathway→ FOXO3↑→ cuproptosis↓ and sunitinib resistant	[90, 92, 95, 106, 107, 109, 112, 113, 123, 146, 147]
NSCLC	LIPT1	DSF→ ATP7B↑ →HIF-1 pathway → PD-L1↑ →tumor immune escape LIPT1↑ → ATOX1↓ → tumor suppression CuET→ bypass GSH→ cuproptosis	[54, 99, 148–150]
BLCA	DLD, PDE3B	PDE3B→KRT6B keratinization and sensitivity of tumor to copper ionophores↑ → tumor suppression GOx@[Cu(tz)] → glucose↓ → cuproptosis sensitivity↑	[115, 151–153]
OS	FDX1, LIPT1, DLAT	T-HCN@CuMS → cuproptosis and cuproptosis sensitivity↑ TFP-Cu → Immune response↑ and cuproptosis→ tumor suppression MCD → ROS↑ → cuproptosis	[91, 157–159]
CCA	FDX1, PDHA1, LIAS, DLD,	CD274↓ → FDX1↓ →cuproptosis sensitivity↑	[90, 106, 109, 115, 160, 161]
OC	MTF1, WASF2	CTC-246B18.8↑ → poor prognosis Anisomycin→ YY1↓ → cuproptosis	[129, 164, 166, 167]
Glioma and GBM	FDX1, PDHA1, DLD, GLS, CDKN2A, SLC31A1	miR-606 → targeting FDX1→ tumor suppression LEF1-AS1↓ → tumor suppression HFn-Cu-REGO NPs → autophagy blockade and cuproptosis → tumor suppression	[90, 106, 115, 123, 126, 168–171, 174]
PDAC	GLS	HBO → efficacy of CuET@PH NPs↑ → tumor suppression	[126, 177, 178]
AML	LIPT1, MTF1, GLS, CDKN2A FDX1, LIAS, DLD, DLAT, PDHA1, SLC31A1, ATP7B	UM4118 → cuproptosis	[89, 90, 115, 123, 181]

### The role of CRGs that promote cuproptosis

#### FDX1

Ferredoxin transports electrons in various biological processes [86]. Human mitochondria contain two ferredoxins, FDX1 (aka adrenergic reduced protein) and FDX2, both of which are indispensable for ubiquitin biosynthesis. FDX1 is involved in the synthesis of sterols, heme a, and lipoyl cofactors. FDX1 and FDX2 have different functions in LIAS-dependent lipoylation. In addition, FDX2 is essential for the maturation of Fe-S proteins [87]. ES can be used to treat copper deficiency and induce cuproptosis to treat cancer. It has been found that FDX1 is uniquely linked to ES. FDX1 plays a key role in releasing copper ions from the ES-copper complexes. Even in the absence of FDX1, copper ions can still be released from the ES-copper complex and utilized outside the mitochondria [88]. In studies [89–91], FDX1 was found to be highly expressed in 14 tumors: colon adenocarcinoma (COAD), diffuse large B-cell lymphoma (DLBC), glioblastoma multiforme (GBM), LUAD, ovarian serous cystadenocarcinoma (OV), pancreatic adenocarcinoma (PAAD), prostate adenocarcinoma (PRAD), rectal adenocarcinoma (READ), skin cutaneous melanoma (SKCM), stomach adenocarcinoma (STAD), thymoma (THYM), uterine corpus endometrial carcinoma (UCEC), osteosarcoma (OS) and uterine carcinosarcoma (UCS), while is lowly expressed in 12 tumors: acute myeloid leukemia (AML), ACC, breast invasive carcinoma (BRCA), cholangiocarcinoma (CCA), head and neck squamous cell carcinoma (HNSCC), kidney chromophobe (KICH), ccRCC, kidney renal papillary cell carcinoma (KIRP), lung squamous cell carcinoma (LUSC), pheochromocytoma and paraganglioma (PCPG), testicular germ cell tumor (TGCT), and thyroid cancer (THCA). In addition, FDX1 is positively correlated with the immune-related gene CD274, and studies have also found that low expression of FDX1 was associated with progression, poor prognosis, and dysregulated immune cell infiltration in ccRCC [92], and high expression of FDX1 was associated with a better prognosis in COAD patients [93]. Therefore, FDX1 could be an important target for tumor immunotherapy.

#### DLAT

The main function of DLAT is to participate in the TCA cycle [94]. In addition, DLAT is involved in other metabolic pathways, such as fatty acid synthesis and amino acid metabolism. The expression of DLAT is up-regulated in LIHC, LUAD, LUSC, OS, and STAD, while it is down-regulated in HNSCC and ccRCC [91, 95]. Fang et al. found that the high expression of DLAT is associated with poor prognosis associated with PAAD patients and increased resistance to commonly used chemotherapeutic agents [96]. In addition, DLAT expression was positively correlated with the infiltration abundance of B cells, CD8^+^T cells, and macrophages. Thus, DLAT expression is closely associated with cancer progression, treatment response, and immune infiltration.

#### Lipoyltransferase 1 (LIPT1)

LIPT1 is an enzyme that plays an important role in embryonic development. Its main function is to transfer the essential coenzyme lipoic acid to the mitochondrial 2-keto acid dehydrogenases associated with the TCA cycle [97]. These enzymes play an important catalytic role in the TCA cycle. Defects in LIPT1 lead to disruption of the TCA cycle, especially the function of alpha-ketoglutarate dehydrogenase (AKGDH) is affected [98]. The expression of the LIPT1 was reduced in NSCLC, and elevated LIPT1 levels were associated with good prognosis in NSCLC patients [99]. It has been found that the expression level of LIPT1 is high in OS [91] and AML [89], the low expression of LIPT1 is associated with poor prognosis in AML, in HCC, the expression level of LIPT1 is also high, and LIPT1 can promote the proliferation and migration of HCC cells and upregulation of LIPT1 expression is associated with poor prognosis in HCC [100, 101], and in melanoma, the upregulation of LIPT1 is positively correlated with PD-L1 expression, additionally, in melanoma patients, melanoma patients with higher levels of LIPT1 expression showed better prognosis [102].

#### Pyruvate dehydrogenase E1 component subunit alpha (PDHA1)

PDHA1 is a subunit of the mitochondrial TCA cycle enzyme pyruvate dehydrogenase complex, which is involved in the conversion of pyruvate to acetyl-coenzyme A (acetyl-CoA). Acetyl-CoA is an important substrate for histone lactylation and the TCA cycle, so PDHA1 is involved in a key step in cellular energy metabolism and also promotes histone lactylation, which in turn affects the expression of pluripotency-related genes [103, 104]. It was found that hyperacetylation and inactivation of PDHA1 led to the overproduction of lactate, which further exacerbated the development of sepsis-induced acute kidney injury (SAKI) through lactation modification [105]. In addition, PDHA1 is differentially expressed in cancers. PDHA1 expression was significantly up-regulated in cervical squamous cell carcinoma (CSCC), cervical squamous cell carcinoma and endocervical adenocarcinoma (CESC), CCA, HCC, LUAD, LUSC, STAD, and UCEC. It was significantly down-regulated in BRCA, GBM, ccRCC, KIRP, PCPG, AML, and THCA. High PDHA1 levels in patients with LUAD were significantly associated with poor prognosis [89, 106]. In ccRCC, PDHA1 expression levels were down-regulated and correlated with a poor prognosis. High expression of PDHA1 is usually associated with poor prognosis in prostate cancer (PCA) [107]. PDHA1 is a downstream target gene, and the stability of its mRNA is regulated by m6A-modified circRBM33, downregulation of circRBM33 decreases ATP production, acetyl-CoA levels, and nicotinamide adenine dinucleotide (NADH/NAD^+^) ratio, thereby inhibiting PCA growth and invasion [108].

#### LIAS

LIAS is involved in the insertion of S atoms during the metabolism of lipoic acid [97]. LIAS plays a catalytic and regulatory role in the Fe-S cluster biosynthetic pathway. Fe-S clusters are important cofactors involved in a variety of enzyme-catalyzed reactions, including fatty acid metabolism, energy production, and DNA repair. In addition, LIAS has a direct binding interaction with the FDX1 protein, which helps to regulate the fatty acylation of proteins in cells [78]. LIAS plays an important role in cancer. It has been found that the expression level of LIAS correlates with the prognosis of a variety of cancers [109]. LIAS expression was up-regulated in CCA, HCC, LUAD, DLBC, THYM, and LUSC, on the contrary, LIAS expression was down-regulated in the majority of cancers, such as BRCA, COAD, ccRCC, KIRP, PRAD, READ, THCA and UCEC. High expression of LIAS was associated with good prognosis in patients with ccRCC, READ, BRCA, and OC, whereas high expression of LIAS was associated with poor prognosis in lung cancer patients.

#### Pyruvate dehydrogenase E1 subunit beta (PDHB)

PDHB is a major subunit of pyruvate dehydrogenase (PDH). PDH catalyzes pyruvate decarboxylation and participates in the glycolytic pathway, thereby regulating cellular energy metabolism [110]. Studies have shown that PDHB activity accelerates tumor cell growth, and overexpression of PDHB can inhibit tumor cell migration and growth by suppressing the signaling pathway [111]. PDHB is lowly expressed in most cancers, such as COAD, HNSCC, ccRCC, KIRP, LUSC, READ, and THCA. And PDHB was highly expressed in HCC. In ccRCC, the expression of PDHB was negatively correlated with the expression of immune checkpoint-related genes, low expression of PDHB may be associated with a better prognosis [112, 113].

#### DLD

DLD participates in the TCA cycle, reducing dihydrolipoic acid produced by the dehydrogenase complex to lipoic acid, as well as reducing NAD^+^ to NADH [97]. DLD is involved in regulating the regulation of a variety of metabolic pathways, including glucose metabolism, fatty acid metabolism, and amino acid metabolism. Abnormal function of DLD may lead to cellular oxidative stress and metabolic disorders, which may trigger the development of diseases [114]. A study found that DLD was lowly expressed in ACC, bladder cancer (BLCA), ccRCC, AML, PCPG, and THCA, and highly expressed in BRCA, CCA, DLBC, GBM, KICH, KIRP, HCC, LUAD, LUSC, PAAD, PRAD, READ, SKCM, STAD, TGCT, and THYM, in BLCA and UCS, high expression of DLD predicted poor prognosis, but upregulation of DLD expression had better prognostic value for ccRCC and KIRP. Moreover, dysregulation of DLD may lead to antitumor drug resistance. The risk of resistance to BMS-690,514 and sarcatinib increased with higher DLD expression levels [115].

#### ZnT1

ZnT1 plays an important role in the cell, not only responsible for transporting Zn from inside the cell to outside the cell, but also involved in copper uptake. A recent study has shown that ZnT1 plays a crucial role in the cuproptosis process [15]. Specifically, ZnT1 is an essential Cu^2+^ import protein that mediates the entry of Cu^2+^ into cells, a process that is necessary for the occurrence of cuproptosis. At the molecular level, Cu^2+^ and Zn^2+^ share the same major binding site in ZnT1, which means that Zn^2+^ can compete with ZnT1-mediated Cu^2+^ uptake. ZnT1 has a unique property in the Zinc transporter family in that it possesses a unique inter-subunit disulfide bond that helps stabilize the outgoing open conformation of both prototypes, thus facilitating efficient Cu^2+^ transport. Overexpression of ZnT1 often predicts a poor prognosis, with significantly elevated ZnT1 mRNA levels in esophageal cancer (ESCA), PAAD, READ, STAD, THYM, and significantly reduced survival compared to controls with low ZnT1 levels [116]. However, HCC is associated with zinc deficiency, and low ZnT1 expression in HCC patients is associated with shorter survival times [117].

### The role of CRGs that inhibit cuproptosis

#### Cyclin dependent kinase inhibitor 2 A (CDKN2A)

CDKN2A is a tumor suppressor gene that encodes two tumor suppressor proteins, p16 and p14, p16 protein’s main function is to inhibit the activity of CDK4 and CDK6, thereby preventing the phosphorylation of Rb and lead to cell cycle arrest between G1/S phases [118, 119], p14 protein, on the other hand, acts by antagonizing MDM2-mediated ubiquitination and degradation of p53 [120]. This prevents excessive proliferation of abnormal cells and tumor formation. Cheng et al. found that CDKN2A upregulates glycolysis, copper metabolism, and copper ion efflux through transcriptional activation of the MEF2D and SNHG7/miR-133b axes thereby inhibiting cuproptosis, while CDKN2A may drive EMT and progression through activation of Wnt signaling [121]. Mutations or deletions in the CDKN2A gene are closely associated with the development and progression of several tumors, including HCC. In HCC, mutation or deletion of CDKN2A leads to cell cycle disorders and unlimited proliferation of tumor cells [122]. CDKN2A expression was significantly up-regulated in ACC, CESC, AML, DLBC, OV, PAAD, PCPG, sarcoma, THYM, and UCS, whereas significantly down-regulated in TGCT. In addition, high CDKN2A expression predicted poor prognosis of ACC, COAD, ccRCC, HCC, PRAD, SKCM, THCA, and UCEC. However, high CDKN2A predicted a better prognosis for GBM patients [123].

#### Glutaminase (GLS)

GLS plays an important regulatory role in cells and is involved in amino acid metabolism and nitrogen metabolism processes. After glutamine enters the cell, it is converted to glutamate under the action of GLS, and glutamate can participate in the synthesis of GSH in addition to turning into α-ketoglutarate acid under the catalytic action of glutamate dehydrogenase to participate in the TCA cycle [124], and GSH, as an intracellular antioxidant, binds to the free Cu ions and reduces the intracellular Cu concentration, thus inhibiting cuproptosis. Zhang et al. reported for the first time a nanomedicine that enhances cuproptosis and immunotherapy by inhibiting GLS. The copper-based nanomedicine (PCB), containing an internal BPTES and copper nanoparticles (Cu-NPs) core as well as an external platelet membrane (PM), contributed to the specific recognition of CD44 proteins highly expressed in tumor cells after intravesical administration thereby targeting the tumor site. The BPTES efficiently inhibited the activity of GLS, which resulted in the reduction of GSH content. Meanwhile, Cu-NPs can cause intracellular oxidative stress and release Cu^2+^ to induce DLAT oligomerization, leading to cuproptosis. PCB inhibits not only primary tumors but also distal tumors. This therapeutic strategy combining GLS inhibition with induction of copper toxicity and enhancement of the immune “cold” environment provides a new way of thinking about the use of cuproptosis to treat tumors [125]. GLS is highly expressed in certain cancer cells [89], such as AML, GBM, breast cancer (luminal), lung cancer and pancreatic ductal adenocarcinoma (PDAC), where it provides glutamine required by tumor cells and promotes tumor cell growth and proliferation, but GLS is lowly expressed in BC (basal) [126]. The researchers found that by transfecting HCC cell lines HepG2 and Hep3B, the up-regulated expression of GLS significantly enhanced the proliferation and metastatic ability and ATPase activity of HCC cells and inhibited cuproptosis [122].

#### Metal-regulatory transcription factor 1 (MTF1)

MTF1 regulates metal ion metabolism and homeostasis in cells. It is activated when the intracellular copper concentration increases and binds to specific DNA sequences, thereby promoting copper transport, storage, and excretion. On the contrary, when intracellular copper concentration decreases, MTF1 activity is diminished, leading to downregulation of the expression of related genes to maintain copper homeostasis [127]. The previously mentioned zinc transporter protein ZNT1 not only excretes zinc but also transports copper into the cell. One study found that knockdown of ZNT1 increased intracellular zinc levels, which in turn activated MTF1 and promoted MT1X expression by strongly driving MTF1 transcription factor. As a result, the interaction between MT1X and copper is enhanced, reducing copper entry into mitochondria and thus inhibiting cuproptosis [128]. The MTF1-MT1X axis also proves that ZnT1 is required for cuproptosis to occur. MTF1 was found to have tumorigenic effects, and knockdown of MTF1 inhibited OC metastasis. Specifically, the knockdown of MTF1 upregulated the expression of KLF4 transcription factor and attenuated two cell survival pathways, ERK1/2 and AKT thereby inhibiting epithelial to mesenchymal transition (EMT), which contributes to the metastasis of OC. The expression of MTF1 was upregulated in OC, and its high expression was correlated with poor survival and disease recurrence in patients [129]. MTF1 is highly expressed in AML, which is associated with poor prognosis [89]. In addition, MTF1 expression is lower in gastric cancer (GC) and is associated with a better prognosis [130].

### Cuproptosis in cancers (Fig. 3)

#### Cuproptosis in HCC

Cuproptosis plays an important role in the progression of HCC. Intratumor Cu and CRGs regulate the expression of immune checkpoints such as programmed cell death protein 1 (PD-1), PD-L1, and cytotoxic T lymphocyte antigen 4 (CTLA-4). Specifically, Wang et al. found that the expression levels of the immune checkpoint genes (ICGs) PDCD1 (the gene encoding PD-1) and CTLA-4 were significantly higher in HCC samples compared to normal liver tissues, and the high expression levels of ICGs in HCC patients may be correlated with poorer prognosis. The expression of the CRGs ATP7A, ATP13A2, SNCA, and PRNP were significantly positively correlated with the expression of ICGs PDCD1, PD-L1, and CTLA-4. However, three ICGs were negatively correlated with CRGs COX17 or F5 [132]. In another study on HCC, the expression of CRGs CDKN2A and GLS was found to be significantly positively correlated with the immune checkpoints PD-L1 and CTLA-4, whereas CRGs DLAT, DLST, and PDHA1 were significantly positively correlated with CD274 only. Taken together, CRGs may be involved in the regulation of ICG expression and tumor immune escape and influence the prognosis of HCC [133]. Li et al. found that maternal embryonic leucine zipper kinase (MELK) enhanced the levels of the CRG DLAT by activation of the PI3K/mTOR signaling pathway thereby promoting the ES drug-resistant and altering mitochondrial function and ultimately accelerating the progression of HCC [134]. ARID1A is among the most commonly mutated tumor suppressor genes in HCC. Xing et al. found that ARID1A-deficient HCC cells and xenograft tumors were highly sensitive to copper treatment, this suggests that copper therapy is a promising therapeutic strategy for selectively targeting ARID1A-deficient HCC [135].

#### Cuproptosis in HNSCC

Cuproptosis may significantly affect the prognosis and clinical features of HNSCC by regulating the TME. Yang et al. analyzed the data of HNSCC patients and established a risk model based on 7 cuproptosis-related long noncoding RNAs (lncRNAs) [136]. The model was able to effectively predict the survival rate of HNSCC patients, and there was a significant difference in the survival rate between the high-risk and low-risk groups. OLR1 is a protein that is closely related to the development of cancer. Wu et al. found that, in HNSCC, OLR1 knockdown enhances oligomerization of DLAT after ES treatment, thereby promoting cuproptosis. In addition, the role of OLR1 may also affect the sensitivity of tumor cells to copper tumors, especially when ES is used [137]. AURKA is an important protein kinase in the family of polarized kinases, and its aberrant expression or mutation is closely associated with the onset and progression of many cancers. Jia et al. found that the knockdown of CRG AURKA significantly inhibited the proliferation and migration of HNSCC cells proliferation and migration. Therefore, CRG AURKA may serve as a potential biomarker for HNSCC [138].

#### Cuproptosis in GC

The study finds cuproptosis associated with prognosis in GC. The N6-methyladenosine (m6A) RNA methyltransferase METTL16 has an important role in human cells [139]. Sun et al. found that METTL16 undergoes lactylation in response to high copper stress and subsequently mediates cuproptosis in GC by up-regulating FDX1 protein expression via m6A modification, resulting in anticancer effects. Therefore, cuproptosis induction becomes a promising therapeutic strategy for GC [140]. CRG serine protease inhibitor clade E member 1 (SERPINE1) is highly expressed in GC and correlates with poor prognosis. SERPINE1 was negatively correlated with CRGs FDX1, LIAS, LIPT1, and PDHA1, and positively correlated with APOE. In addition, SERPINE1 was associated with immune infiltration and positively correlated with resting natural killer (NK) cells, neutrophils, activated mast cells, and macrophage M2, while negatively correlated with B cell memory and plasma cells [141].

#### Cuproptosis in colorectal cancer (CRC)

Recently, the role of cuproptosis in CRC has attracted great interest from researchers. It was found that most CRGs were differentially expressed between CRC tissues and normal tissues and that a risk scoring system based on CRGs had a good predictive value for the prognosis of CRC patients as well as a reference value for immunotherapy [142, 143]. 4-octyl itaconate (4-OI, a cell-permeable itaconate derivative) has excellent anti-inflammatory and antioxidant properties [144]. Yang et al. found that FDX1 knockdown impaired the ability of 4-OI to promote cuproptosis. In in vivo experiments, 4-OI with ES-Cu showed better anti-tumor effects. This suggests that ES-Cu rapidly stops cell growth in CRC cells and oxaliplatin-resistant cell lines. Additionally, 4-OI inhibits aerobic glycolysis by targeting glyceraldehyde 3-phosphate dehydrogenase (GAPDH) to promote cuproptosis and exerts anti-inflammatory and antioxidant effects through activation of nuclear factor erythroid 2-related factor 2 (Nrf2), which promotes CRC cell death [145]. These may be effective treatments for CRC.

#### Cuproptosis in ccRCC

CRGs affect ccRCC progression and prognosis. PDHB is one of the CRGs that can inhibit the proliferation, migration, and invasion of ccRCC cells [112]. In ccRCC, the expression of FDX1 was significantly lower than that in normal tissues, and the low expression of FDX1 was in turn associated with progression, poor prognosis, and dysregulated immune cell infiltration in ccRCC [92]. Xie et al. identified FDX1 as a tumor suppressor in ccRCC and characterized the miR-21-5p/FDX1 axis in ccRCC. MiR-21-5p acted as an upstream regulator of FDX1 to drive the development of ccRCC, and promoted the growth and invasive ability of tumor cells by inhibiting FDX1 expression. In addition, FDX1 expression was positively correlated with the CD4^+^ T cell population, and the T cell immune response may be associated with FDX1-mediated tumor suppression in ccRCC. These suggest that this axis may open up new horizons for the treatment of ccRCC [146]. Wang et al. found that adrenomedullin (ADM) has been implicated in drug resistance to sunitinib, a commonly used targeted therapy, in ccRCC. Upregulation of ADM activates the p38/MAPK pathway, which facilitates phosphorylation of forkhead box O3 (FOXO3) and its entry into the nucleus. Increased nuclear FOXO3 inhibits FDX1 transcription, which inhibits cuproptosis and thus promotes drug resistance [147].

#### Cuproptosis in NSCLC

Studies focusing on the role of cuproptosis in NSCLC are increasing. DSF exerts anti-tumor effects through cuproptosis. Li et al. found that DSF activates the HIF-1 signaling pathway by up-regulating the expression of ATP7B, which induces the upregulation of PD-L1 expression and enhances the immunosuppressive and immune escape effects in NSCLC. Therefore, the resistance of NSCLC to chemotherapy can be overcome by the combined application of DSF and anti-PD-L1 for the treatment of NSCLC [148]. Cisplatin is a widely used drug in cancer chemotherapy, but the anti-tumor effect of cisplatin is limited by the elevated concentration of GSH in tumor cells, which leads to the development of chemotherapy resistance [54]. Lu et al. found that copper(II) bis(diethyldithiocarbamate) CuET, a copper-based nanomedicine, bypasses GSH interference and exerts antitumor effects on NSCLC cells as well as reverses cisplatin resistance via the mechanism of cuproptosis [149]. Deng et al. found that expression of the CRG LIPT1 was reduced in NSCLC, and elevated LIPT1 levels were associated with favorable prognosis in NSCLC patients. Specifically, LIPT1 overexpression inhibited NSCLC cell proliferation by down-regulating ATOX1 under copper-stimulated conditions [99]. Furthermore, Tang et al. constructed a risk scoring model for CRGs that can better predict prognosis and immunotherapy response in NSCLC patients [150]. In conclusion, these findings provide new potential targets and strategies for individualized treatment and immunotherapy of NSCLC.

#### Cuproptosis in BLCA

The treatment and role of cuproptosis in BLCA sparks research interest. Xu et al. developed a glucose oxidase (GOx)-engineered nonporous copper(I) 1,2,4-triazolate ([Cu(tz)]) coordination polymer (CP) nanoplatform, denoted as GOx@[Cu(tz)], which can be activated by GSH stimulation in tumor cells, thus reducing glucose levels in tumor cells. This starvation therapy can make tumor cells more sensitive to copper-induced toxicity, thereby inducing cuproptosis for anticancer effect. It was demonstrated that this nanoplatform had a significant inhibitory effect on the growth of BLCA in mice without inducing significant systemic toxicity [151]. Guo et al. designed a ROS-sensitive polymer (PHPM), which was used to co-encapsulate ES and Cu to form nanoparticles (NP@ESCu). In in vivo experiments, NP@ESCu was found to be able to induce cuproptosis and remodel the TME in a subcutaneous BLCA mouse model [152]. Feng et al. identified phosphodiesterase 3B (PDE3B), a CRG, which reduces the invasion and migration of BLCA. The anticancer effects of PDE3B are mediated by causing keratinization of keratin 6B (KRT6B). PDE3B activation increases the sensitivity of BLCA cells to copper ionophores, and PDE3B/KRT6B is a potential target for cancer therapy [153]. Wang et al. developed a new copper intoxication-associated lncRNA risk model that predicts outcome and immunotherapy response [154]. Song et al. established a cuproptosis scoring system to predict the clinical outcome and immune response in BLCA [155]. These studies could provide new insights into prognostic assessment and potentially guide comprehensive treatment of BLCA.

#### Cuproptosis in OS

Research utilizing cuproptosis to treat OS is increasing. The expression of FDX1, DLAT, LIPT1 was significantly upregulated in OS [91]. Jia et al. successfully developed an OS risk model based on cuproptosis and mitochondria-associated markers with strong predictive power in terms of prognosis and immune landscape [156]. Xia et al. developed a novel nanomaterial, the heterogeneous carbon nitride-based nanoagent named T-HCN@CuMS, which exhibits excellent anti-tumor effects on OS cells under near-infrared light irradiation. The nanomaterial achieves anti-tumor therapy by inducing intracellular oxidative stress and cuproptosis and further enhancing cellular sensitivity to cuproptosis [157]. Xie et al. designed Cu ion-coordinated Tremella fuciformis polysaccharide (TFP-Cu), which induces cuproptosis by releasing copper ions for the treatment of OS and activates immune cells to enhance the immune response, with inhibitory effects on tumor growth and proliferation [158]. Furthermore, a novel material, GSH and pH-responsive organic-inorganic mesoporous silica nanoparticles@Cu2S@oxidized Dextran (short for MCD), is used to treat OS. Specifically, MCD affects proteins such as DLAT and LIAS, which in turn affect the TCA cycle, leading to OS cell death via cuproptosis. Meanwhile, the material produces ROS, which further facilitates this process [159].

#### Cuproptosis in CCA

CRGs have an important role in CCA. Changes in CRGs signature expression can predict the clinical prognosis of CCA fairly accurately [160]. Shen et al. knocked down the CD274 gene in ICC cells by transfection of siRNA and found that the expression level of FDX1 in the cells was significantly downregulated. These cells were more prone to cuproptosis after the addition of ES-Cu [161]. He et al. developed a CRGs scoring system that accurately predicts prognosis, as well as initially identified glycine cleavage system protein H (GCSH), a cuproptosis key gene, as a reliable therapeutic target or prognostic indicator for CCA patients [162]. Yao et al. investigated the role of cuproptosis-related lncRNAs (CRLs) in prognostic prediction and immune infiltration in CCA. They established a risk marker that can be used as an independent prognostic factor to predict not only the prognosis but also the tumor immune microenvironment (TIME) of patients with CCA [163]. These findings help us to better understand the pathogenesis of ICC and provide new ideas for future therapeutic strategies.

#### Cuproptosis in OC

CRGs are a key link between cuproptosis and OC prognosis, a study showed that CRG WASP family member 2 (WASF2) promotes cancer cell proliferation and platinum resistance, and its high expression is associated with poor prognosis in OC patients [164]. High expression of CRL CTC-246B18.8 is associated with poor prognosis and an immunosuppressive TME and is a promising prognostic biomarker and therapeutic target for OC [165]. Nie et al. found that anisomycin exerts antitumor effects by triggering cuproptosis through inhibition of transcription factor YY1 expression and activity in OC stem cells [166]. It was found that in OC cells, DSF could inhibit cell proliferation and promote apoptosis by inducing cuproptosis [167]. In addition, cuproptosis can enhance the immune response by interfering with the metabolism of copper ions. However, further studies are needed to validate the efficacy and safety of DSF in the treatment of OC. These findings provide a theoretical basis for the use of cuproptosis as an adjuvant therapy for OC and provide new targets for the development of related drugs.

#### Cuproptosis in glioma

The expression of CRGs correlates with the immune microenvironment, pathological grading, and prognosis of gliomas [168, 169]. Zhang et al. found that miR-606 inhibited the glycolysis and proliferative capacity of glioma cells by targeting the 3’UTR region of the FDX1 gene [170]. There is also a study that constructed a CRGs-signature for distinguishing different copper homeostatic features of glioma patients, accurately predicting the clinical characteristics of glioma patients, and providing guidance for cuproptosis treatment targeting glioma [171]. Meanwhile, the CRL signature is also a prognostic and treatment response indicator for glioma patients. Inhibition of LEF1-AS1 prevented glioma growth, migration, and invasion. LEF1-AS1 may be an effective prognostic biomarker and therapeutic approach for glioma [172]. GBM is the most malignant form of glioma with an extremely poor prognosis. The Cuproptosis Activation Scoring (CuAS) Model established by Zhou et al. has stable and independent prognostic efficacy. Epiregulin (EREG), is a core tumor immune biomarker in CuAS. The expression level of EREG was positively correlated with the expression of PD-L1 and negatively correlated with the expression of FDX1. So EREG has immunotherapeutic potential by affecting PD-L1 expression and anti-tumor potential by mediating cuproptosis through affecting FDX1 expression. In addition, EREG modulates the interactions of vascular endothelial growth factor (VEGF) and CD99 signaling in glioblastoma, providing support for immunotherapy and chemotherapy [173]. Jia et al. designed a biomimetic nanoplatform (HFn-Cu-REGO NPs) consisting of Cu^2+^, the chemotherapeutic drug regorafenib, and human H-ferritin (HFn) capable of traversing the blood-brain barrier and targeting tumors. HFn possesses the ability to penetrate the blood-brain barrier (BBB) and target tumors through interaction with transferrin receptor 1 (TfR1). Cu^2+^ promotes the encapsulation of regorafenib with HFn through its binding to the metal-binding sites and promotes the encapsulation of regorafenib with HFn. Regorafenib can effectively inhibit autophagosome-lysosome fusion, leading to lethal autophagy blockade, and Cu^2+^ can interfere with copper homeostasis in GBM cells, triggering cuproptosis. In this way, HFn-Cu-REGO NPs in GBM therapy achieved significant inhibitory effects with little adverse effects on normal tissues, opening new avenues for enhancing GBM therapy by manipulating autophagy and cuproptosis [174].

#### Cuproptosis in PDAC

Cuproptosis may affect the prognosis of PDAC patients by modulating the TIME [175]. PDAC patients can be categorized into different subtypes based on the expression pattern with CRGs. These subtypes differ in biological function, immune cell infiltration and chemotherapy response [176]. It is widely known that nanomedicines targeting cancer stem cell (CSC) should be considered as a promising tool to improve the sensitivity of CSC and the efficacy of specific anti-CSC treatments [177]. Xiao et al. found that hyperbaric oxygen (HBO) modulates CSC metabolism by overcoming tumor hypoxia and enhances the efficacy of polydopamine and hydroxyethyl starch stabilized copper-diethyldithiocarbamate nanoparticles (CuET@PH NPs) in eliminating CSCs, resulting in potent tumor suppression of PDAC [178]. These findings provide new clues and strategies for individualized treatment of PDAC.

Cuproptosis in AML.

CRGs are differentially expressed in AML patients and are highly correlated with poor prognosis, CRGs LIPT1, MTF1, GLS, and CDKN2A are highly expressed in AML, whereas FDX1, LIAS, DLD, DLAT, PDHA1, SLC31A1, and ATP7B are lowly expressed in AML, high expression of MTF1 and low expression of LIPT1 are associated with poorer prognosis in AML patients [89]. The CRL signature established by Li et al. and risk markers established by Cao et al. can be used as reliable biomarkers of AML prognosis to inform potential therapeutic strategies [179, 180]. In addition, Moison et al. developed a copper ion carrier called UM4118, which can induce cuproptosis in AML more efficiently, providing a new approach to the treatment of AML [181].

## Copper and cuproptosis in TME

**Fig. 4:**
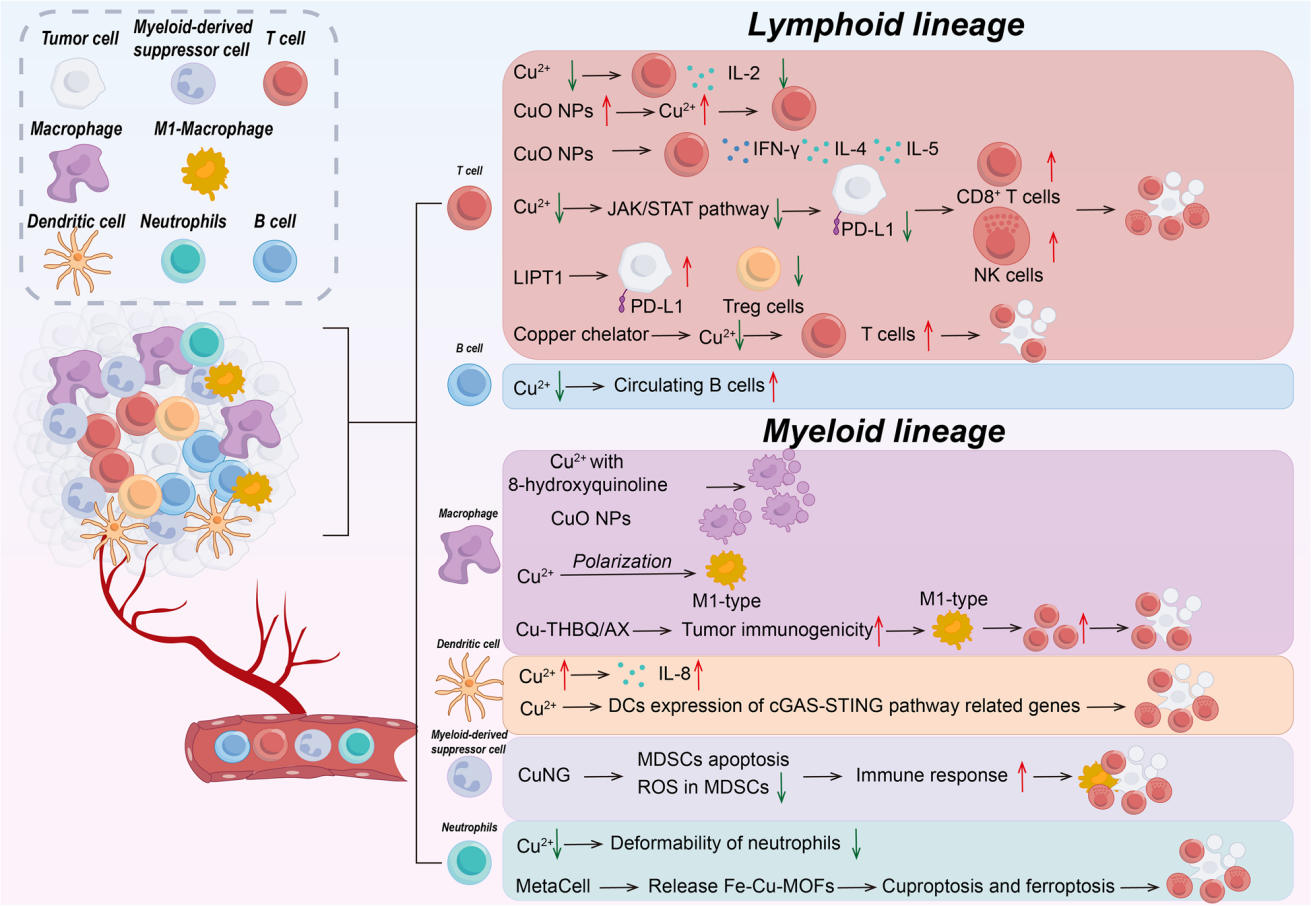
Copper and cuproptosis in immune cell biology

**Fig. 5:**
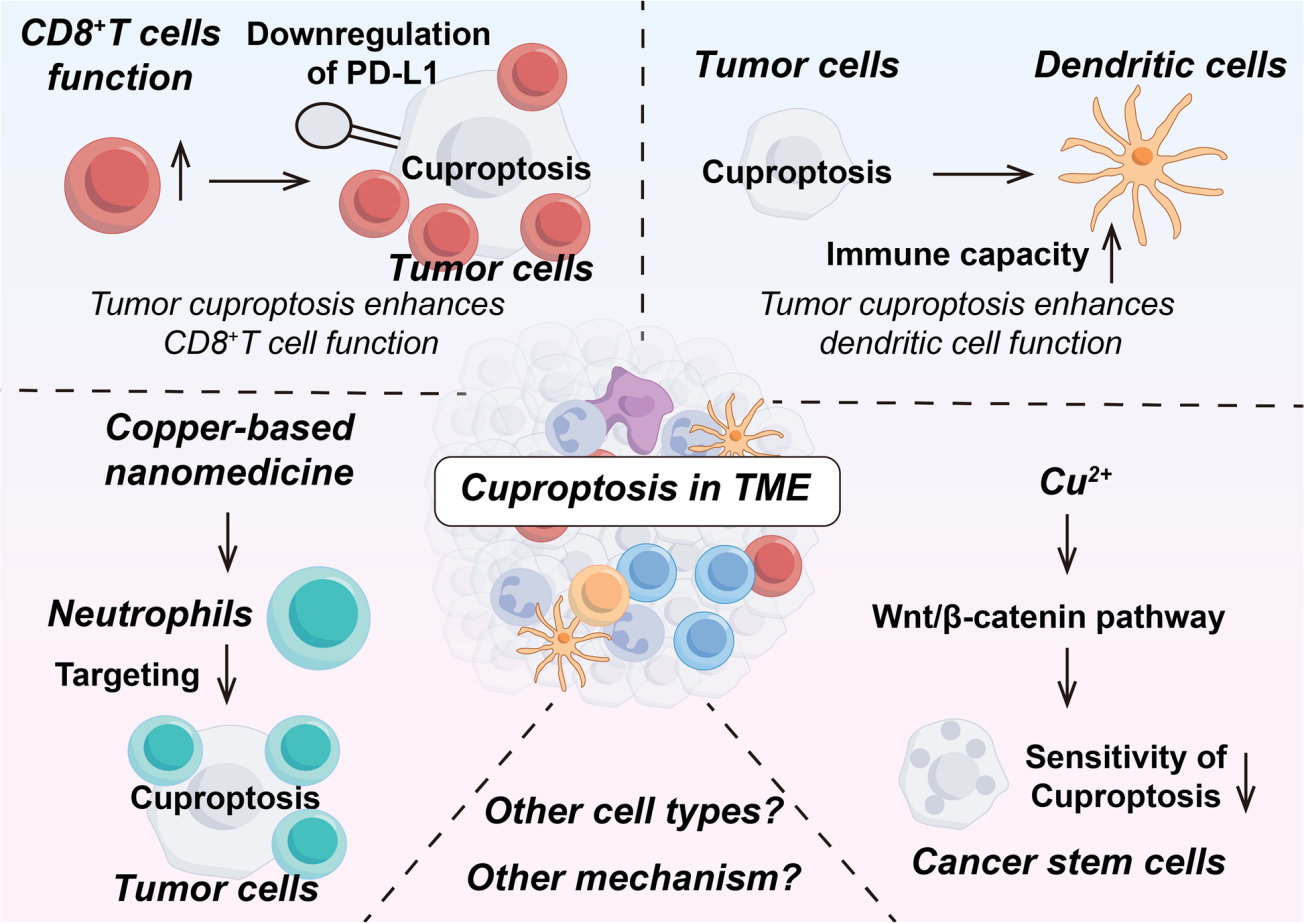
The role of cuproptosis within the TME and its interaction with immune cells

The immune system consists of immune organs, immune cells, and immune molecules, and is the most effective weapon of the body to defend against tumors [182]. TME refers to the microenvironment around the tumor cells, including the tumor cells themselves, and non-tumor cellular components such as a variety of immune cells, mesenchymal stromal cells, and the neovascularization system. Tumor and TME are closely linked, and tumors can affect TME by releasing various cell signaling molecules, and similarly immune cells in TME can affect tumor growth and development [183]. Regarding tumor therapy, the focus has shifted from the tumor-centered approach to the TME-centered one, and various drugs targeting the TME have emerged in an endless stream [184]. Here, we will make the following subdivision of copper and cuproptosis in regulating the TME (Tables 3 and 4; Figs. 4 and 5).

**Table 3:** Cu and cuproptosis in immune cell biology

Immune cells		Cu in immune cell	Cu in tumor immune	Ref.
Lymphoid lineage	T cell	Cu↓ → IL-2↓ CuO NPs → Cu↑ → T cell↑ CuO NPs → IFN-γ, IL-4, IL-5↑	Cu↓ → JAK/STAT pathway↓ → PD-L1↓ → CD8^+^T cell and NK cell↑ → tumor suppression copper chelator→ Cu↓→ T cell↑→ tumor suppression LIPT1↑ → PD-L1↑and Treg↓	[102, 185–195]
	B cell	Cu↓→circulating B cells↑	**\**	[223]
Myeloid lineage	Macrophage	Cu with 8-hydroxyquinoline → macrophage apoptosis Cu → macrophage polarization → pro-inflammatory M1-type CuO NPs → macrophage death CTMM → regulate macrophage, cuproptosis and ferroptosis →eliminate intracellular infection	Cu-THBQ/AX → tumor immunogenicity↑ → polarize M2-type to M1-type macrophages and T cell↑ → tumor suppression	[197–208]
	DC	Cu → IL-8↑	Cu→DCs expression of cGAS-STING pathway related genes↑→tumor suppression	[219–222]
	MDSC	**\**	CuNG → MDSCs apoptosis ↑and ROS in MDSCs↓ → immune response↑ → tumor suppression	[216–218]
	Neutrophil	Cu↓ → Neutrophils↓ and deformability of neutrophils↓	MetaCell → release Fe-Cu-MOFs → cuproptosis and ferroptosis → tumor suppression	[209–215]

**Table 4:** Copper-based nanomedicines in cancer treatment

Copper-based nanomedicines	Mechanism of anti-tumor	References
PCB	Induce cuproptosis	[125]
PHPM	Induce cuproptosis and remodel TME	[152]
MCD	Induce cuproptosis	[159]
CuET@PH NPs	Suppresses energy metabolism of CSCs and induce cuproptosis	[178]
DSF/MS-Cu-2	Enhance efficacy of anti-CTLA-4 antibody	[237]
G5-PBA@CuS/cGAMP	Photothermal-triggered anti-tumor immune	[243]
Cu-THBQ/AX	Enhance anti-tumor immune	[208]
ES-Cu-MOF	Induce cuproptosis and ICD	[265]
PDA-DTC/Cu	Induce cuproptosis and ICD	[266]
Cel-Cu NP	Induce cuproptosis and ICD	[267]
PCD@Cu	Induce cuproptosis and ICD	[268]
SPNLCu	Deplete lactate, induce cuproptosis and ICD	[270]
mCGYL-LOx	Deplete lactate, induce cuproptosis and ICD	[271]
CAT-ecSNA-Cu	Alleviate hypoxia, induce cuproptosis and ICD	[272]
BAu-CuNCs	Induce cuproptosis and ICD	[273]
CCNAs	Enhance apoptosis, induce cuproptosis and ICD	[274]
MACuS	Induce cuproptosis and ICD	[275]
CHP	Reprogram TME, induce cuproptosis and ICD	[276]

### T cell

T cells are vital for maintaining good health and are an important part of the adaptive immune system. T cell development occurs in the thymus, where T cells differentiate into predominantly CD4^+^ and CD8^+^ T cell subpopulations. And naïve T cells differentiate upon stimulation into CD4^+^ helper and CD8^+^ cytotoxic effector and memory cells, which allow T cells to defend against pathogens and tumors, thus maintaining immune homeostasis [185]. Copper is important for regulating T cells [186]. Interleukin-2 (IL-2) has a pro-differentiation and pro-proliferative effect on T cells as well as exerting a T cell killing ability [187]. Copper deficiency decreases IL-2 production and IL-2 mRNA levels in human T cells while copper supplementation reverses this change [188, 189]. Copper oxide nanoparticles (CuO NPs) are used in industry and medicine. Inhalation of CuO NPs significantly increased the copper content in the lungs and livers of mice, causing a significant increase in the proliferative response of T cells. IFN-γ, a pro-inflammatory cytokine produced by T cells and NK cells, induces PD-L1 upregulation in cancer cells and enhances immune evasion [190]. In addition, CuO NPs significantly induced the production of Th1 cytokine IFN-γ and Th2 cytokines IL-4 and IL-5 [191].

Copper also has an important role in regulating T cell-mediated tumor immunity. Copper chelators may increase T cell infiltration in tumors by decreasing PD-L1 expression [192]. Specifically, decreased copper levels downregulate the JAK/STAT signaling pathway, which in turn inhibits IFN-γ-stimulated PD-L1 upregulation as well as by inhibiting EGFR signaling and promoting PD-L1 ubiquitination and degradation, which would enhance infiltration of immune cells CD8^+^ T cells and NK cells, thereby increasing tumor mouse survival. Crowe et al. found that mesothelioma growth was inhibited by the use of copper chelators and antibodies targeting VEGF, which reduced copper levels in vivo to normalize tumor vasculature and increase T-cell infiltration [193]. Regulatory T cells (Tregs) belong to the CD4^+^ T cell subset and are immunosuppressive, downregulating or inhibiting the induction and proliferation of effector T cells. In some tumors, Tregs promote the immune escape of tumor cells [194, 195]. It was found that in melanoma, upregulation of the CRG LIPT1 was positively correlated with PD-L1 expression and negatively correlated with Treg infiltration. Tumor response to immunotherapy can be improved by inhibiting Tregs infiltration in TME [102]. Furthermore, a study found that cuproptosis of CRC cells may enhance the immune function of CD8^+^T cells by down-regulating the Wnt signaling pathway and decreasing the expression of PD-L1 [196].

### Macrophages

As an important component of innate immunity, macrophages, most of which are differentiated from bone marrow monocytes, have three basic functions: immunomodulation, phagocytosis and antigen presentation [197]. Tumor-associated macrophages (TAMs) are an important component of TME and play an important role in tumor development and drug resistance by creating an immunosuppressive microenvironment [198]. Macrophages are polarized in different directions according to different stimuli and are divided into classically activated M1-type macrophages (pro-inflammatory) and selectively activated M2-type macrophages (anti-inflammatory) [199]. Copper has been found to induce macrophage apoptosis in the presence of 8-hydroxyquinoline, as well as the copper transporter protein ceruloplasmin exerts a similar effect [200]. According to a study by Zangiabadi et al., copper attenuates the production of inflammatory factors and chemokines produced by macrophages in response to bacterial lipopolysaccharide (LPS) stimulation. In addition, copper inhibited the expression of inflammation-related genes. These results suggest that copper may have an anti-inflammatory effect and that the anti-inflammatory effect correlates with the concentration of copper, as well as modulating the inflammatory response of macrophages [201]. Copper can also polarize macrophages to generate a pro-inflammatory M1-type as well as promote angiogenesis through secretion of VEGF [202, 203]. Copper is a common additive to implant materials, and Wang et al. found that macrophages secreted exosomes to promote angiogenesis in response to copper ion stimulation, which is important in the osseointegration of implants [204]. According to a recent study, Zhang et al. developed an ultrasound-responsive copper-based nanoparticle targeting macrophages by encapsulating copper in a porphyrin metal-organic framework and surface-grafting D-Mannosamine to synthesize CuTCPP@MOF nanodots@Mannosamine (CTMM) with macrophage-targeting functionality. By overloading the CTMM through the action of ultrasound, the CTMM could induce a cupferroptosis-like stress by regulating macrophage oxidative stress metabolism and inducing cuproptosis and ferroptosis simultaneously to eliminate intracellular infection [205]. In addition, CuO NPs can trigger macrophage death and induce misfolding of SOD1. Copper may also affect macrophage function and viability through mechanisms such as oxidative stress. However, further studies are needed to reveal the specific mechanism of action involved [206].

In terms of macrophages against tumors, Lu et al. found that copper affects macrophage response by inducing the release of immunogenic substances from TAMs [207]. Specifically copper-induced damage-associated molecular patterns (DAMPs) attract macrophages to migrate toward tumor cells and increase macrophage polarization. These polarized macrophages can release relevant cytokines that enhance their anti-tumor immunity. Recently, researchers designed a nanovaccine called Cu-THBQ/AX that uses copper to regulate macrophages to kill tumors [208]. Specifically, Cu-THBQ/AX promotes the specific and controlled release of XMD8-92 through a Fenton-like reaction, which inhibits macrophage phagocytosis, thereby effectively inhibiting macrophage phagocytosis of apoptotic cancer cells and converting apoptotic cells into secondary necrosis. In addition, Cu-THBQ/AX could enhance tumor immunogenicity, promote dendritic cell maturation and antigen presentation, polarize M2-type macrophages to pro-inflammatory M1-type macrophages, and effectively infiltrate activated T cells into tumor tissues. These studies open up new horizons for using macrophages to kill tumors.

### Neutrophils

Neutrophils play an important role in immune defense during infection and inflammation, participating in the inflammatory response through chemotaxis, phagocytosis, and production of anti-microbial effectors, and are regulated by anti-inflammatory signals to terminate the inflammatory response [209]. Meanwhile, neutrophils are plastic and can exhibit both pro- and anti-tumor functions in tumor tissues [210]. Copper levels affect the number and function of neutrophils [211, 212]. Copper deficiency causes a decrease in neutrophils [213]. It was found that in copper-deficient rats, neutrophils accumulate in the lung microvasculature and the deformability of neutrophils is diminished [214].

Recently, researchers have developed a new drug, MetaCell, that can be used to treat tumors using neutrophils to induce cuproptosis and ferroptosis [215]. Specifically, MetaCell consists of live neutrophils that encapsulate a thermosensitive liposomal formulation of bimetallic Fe-Cu metal-organic frameworks (Lip@Fe-­Cu-­MOFs). MetaCell exploits the unique properties of neutrophils to evade the immune system, infiltrate tumors, and respond to inflammation. Once internalized, these heat-sensitive liposomes stimulate the targeted release of Fe-Cu-MOFs from cancer cells upon exposure to near-infrared light and enhance the simultaneous activation of cuproptosis and ferroptosis mediated by Fe-Cu-MOFs, thereby exerting a potent anti-tumor effect.

### Myeloid-derived suppressor cells (MDSCs)

Under normal conditions, immature myeloid cells (IMCs) arise in the bone marrow and differentiate into dendritic cells, granulocytes, and macrophages. However, under some pathological conditions such as inflammation, infection, and cancer, IMCs enter the circulatory system directly and help tumor cells evade immune surveillance, which are then called MDSCs. MDSCs are classified into granulocyte-like MDSCs or polymorphonuclear-like MDSCs (G-MDSCs or PMN-MDSCs) and mononuclear phagocyte-like MDSCs (M-MDSCs) [216]. MDSCs aid in cancer immune evasion and make tumors resistant to immunotherapy. Therefore, eliminating MDSCs can improve the effectiveness of cancer treatment and patient survival [217].

It was found that copper N-(2-hydroxy acetophenone) glycinate (CuNG), a copper chelator, induces apoptosis in MDSCs and reduces their numbers in the TME. This leads to the conversion of tumor-associated CD4^+^ T cells to the Th1 type, which enhances the immune response. In addition, CuNG reduces ROS levels in MDSCs, which further enhances their anti-tumor activity. This suggests that the use of copper chelators can disrupt the immunosuppressive network in tumors, thereby improving immune function in tumor patients [218].

### Dendritic cells (DCs)

DCs are an important component of the immune system, acting as a bridge between innate and adaptive immunity. When DCs are stimulated, they release a variety of cytokines, including interleukin-8 (IL-8), a chemokine that attracts other immune cells toward the stimulated area and participates in inflammatory responses and immune responses [219]. One study showed that copper salts were found to significantly increase IL-8 production by measuring the levels of IL-8 released by cells [220]. This suggests that copper may affect DCs activation and inflammatory responses. However, compared to nickel, cobalt and palladium, the stimulatory effect of copper was weak.

In one study, by pretreating tumor cells with cuproptosis-inducing agents such as ES and Cucl2, researchers observed that in a co-culture system of DCs and tumor cells, DCs expression of genes related to the cGAS-STING signaling pathway (an important immune response pathway that can promote anti-tumor immune responses) significantly increased [221]. In addition, the researchers observed that pre-treated tumor cells released more cGAMP, a secondary signaling molecule of cGAS, and that cells in the co-culture system released more immune-associated proteins such as IL-2, TNF-α, IFN-γ, CXCL10, and CXCL11. These results suggest that activation of cuproptosis enhances the interaction between tumor cells and DCs and promotes immune response [222].

### Others

There are relatively few studies on the role of copper in B cells and NK cells. One study showed an increase in the percentage of circulating B cells on a low-copper diet, but the addition of copper to the ration had no significant effect on the number of circulating NK cells [223]. CSCs are also present in TME, which are a part of tumor cells with self-renewal and multiple differentiation potentials, and play an important role in tumor development, metastasis, and treatment resistance [224], while CSCs are also considered as one of the key factors of intra-tumor heterogeneity [225]. A recent study found that copper binds to PDK1 and promotes its interaction with AKT, leading to activation of the Wnt/β-catenin pathway and CSC properties. The aberrant activation of Wnt/β-catenin signaling leads to the upregulation of the β-catenin/TCF4 transcriptional complex by binding directly to the ATP7B promoter, which subsequently inhibits cuproptosis by decreasing copper concentration through the exocytosis of intracellular copper ions [226]. Further research is needed in the future to research the link between copper and cuproptosis in TME.

## Copper and cuproptosis in improving antitumor immunity

**Fig. 6:**
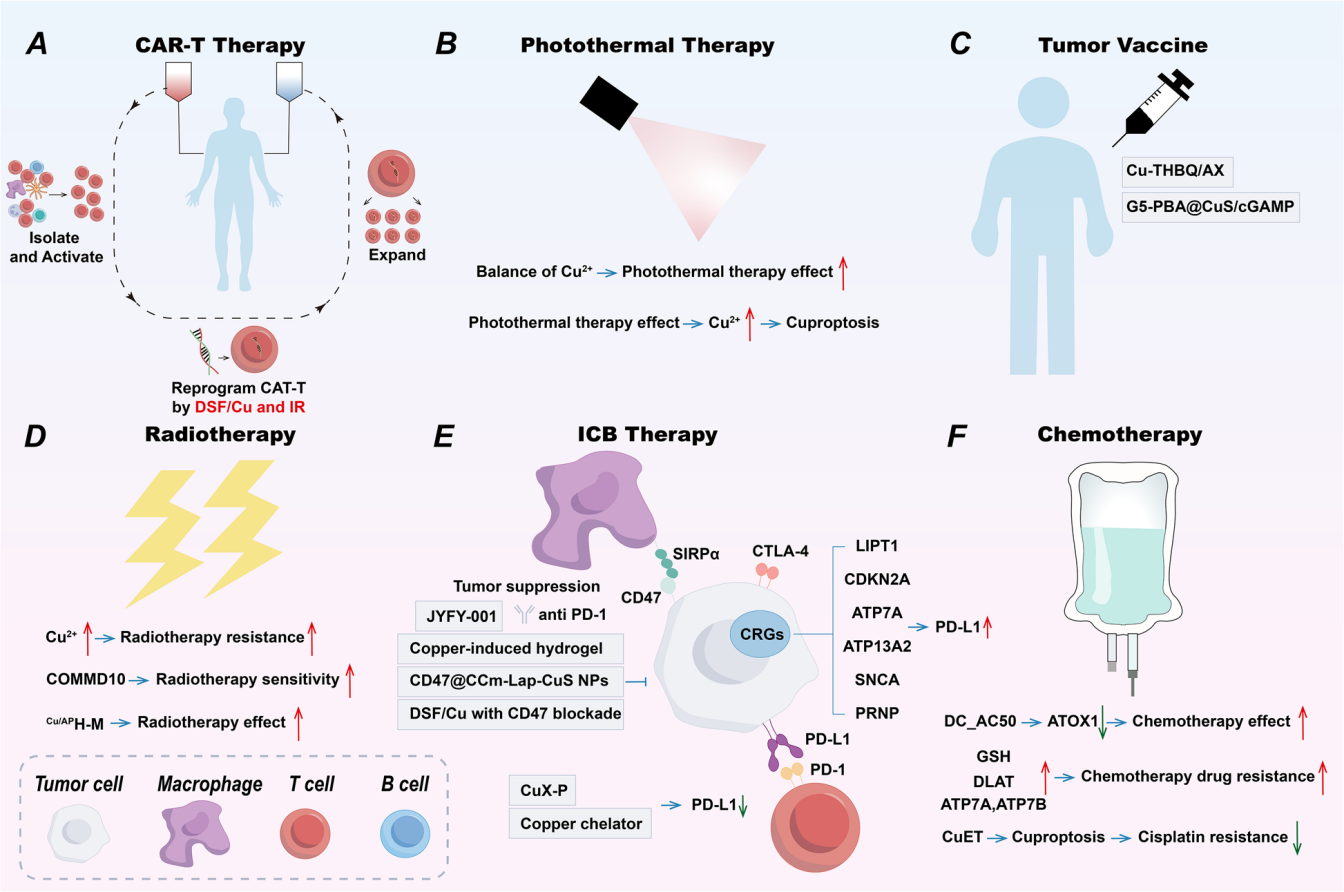
Copper and cuproptosis in cancer treatment

Under normal circumstances, the body’s immune system will identify and remove the tumor cells in the TME. However, the tumor cells are very cunning, in order to their growth and metastasis, the tumor cells will make the body’s immune system inhibit the immune effect on the tumor through a series of mechanisms so that the body cannot be able to kill the tumor through the immune system, this phenomenon is known as the immune escape of the tumor [227]. Recently, immunotherapy for tumors has been developing rapidly, such as immune checkpoint blockade, tumor vaccine, immune cell therapy and other therapeutic means have appeared, which brings new hope for tumor patients. Copper has an important role in boosting anti-tumor immunity, and with the proposal of cuproptosis, a novel copper-induced cell death mechanism, more and more studies have been conducted on using copper to improve immunotherapy for tumors. Here, we will briefly describe the utilization of copper and cuproptosis in four tumor immunotherapies (Table 5; Fig. 6).

**Table 5:** Copper and cuproptosis in cancer treatment

Cancer treatment		Mechanism	Ref.
Tumor immunotherapies	ICB PD-1/PD-L1	LIPT1, CDKN2A, ATP7A, ATP13A2, SNCA, PRNP ↑ → PD-L1↑ DSF → PD-L1↑ Copper chelator → PD-L1↓ CuX-P → PD-L1↓ JYFY-001 with anti-PD-1 → tumor suppression copper-induced hydrogel → anti-PD-L1efficacy↑	[102, 132, 133, 148, 192, 229–232]
	ICB CD47	CD47@CCM-Lap-CuS NPs → tumor suppression DSF/Cu with CD47 blockade → tumor suppression	[233–235]
	ICB CTLA-4	DSF/MS-Cu-2 with anti-CTLA-4 antibody → tumor suppression	[236, 237]
	CAR-T	Reprogram CAR-T by DSF / Cu and IR → solid tumor suppression	[238–240]
	Tumor vaccine	Cu-THBQ/AX → tumor immunogenicity↑ → polarize M2-type to M1-type macrophages and T cell↑ → tumor suppression G5-PBA@CuS/cGAMP → tumor suppression	[208, 241–243]
	Non-specific immune enhancer	Cu regulates immune system Cu NPs enhance immune responses	[244–246]
Other treatment	Copper ionophores ES and DSF	ES and DSF → cuproptosis → tumor suppression	[5, 247, 253]
	Copper chelator	Copper chelator → Cu↓→tumor suppression and chemotherapy sensitivity↑	[259–261]
	Chemotherapy	DC_AC50→ATOX1↓ → chemotherapy effect↑ GSH、DLAT、ATP7A and ATP7B↑ → chemotherapy drug resistance↑ CuET → cuproptosis → cisplatin resistance↓	[35, 54, 60, 61, 96, 149, 259]
	Radiotherapy	Cu↑→ radiotherapy resistance↑ COMMD10 → radiotherapy sensitivity↑ ^Cu/AP^H-M → radiotherapy effect↑	[77, 262, 263]
	Photothermal therapy	Regulate Cu balance → photothermal therapy effect↑→ tumor suppression Photothermal therapy → Cu↑→ cuproptosis → tumor suppression	[65, 230, 232, 234, 243]
	Nano therapy	Copper-based nanomedicines → cuproptosis and improve anti-tumor immune via ICD →tumor suppression	[65, 125, 152, 159, 178, 237, 243, 265–268, 270–276]

### Immune checkpoint blockade (ICB)

The human immune system reduces unnecessary autoimmune responses by immunosuppression through immune checkpoints. Still, in TME, cancer cells use immune checkpoints to evade the immune system. Hence, ICB therapy interferes with receptor-ligand interactions between tumor cells or antigen-presenting cells and T cells to activate T-cell-mediated antitumor responses [228]. Here, we review the role of copper in ICB.

#### PD-1/PD-L1

ICB therapies targeting PD-1/PD-L1 are a hot research topic in tumor immunity [229]. We have mentioned that CRGs modulate immune checkpoint expression in tumors [102, 132, 133], DSF induces upregulation of PD-L1 expression in NSCLC [148], and copper chelators promote PD-L1 ubiquitination and degradation [192]. Recently, Liu et al. developed a tumor-targeting bionanomimetic system for simultaneous cuproptosis killing, photothermal therapy, and immunotherapy in a triple-negative BC mouse model [230]. Specifically, MXene nanosheets loaded with DSF/Cu^2+^ were coated on the membranes of T cells overexpressing PD-1 to generate the CuX-P system. The CuX-P recognized and adhered to PD-L1 on the tumor cells like a patch, promoting endocytosis of CuX-P and PD-L1 by the tumor cells. With the internalization and release of DSF/Cu^2+^ in the cytoplasm, PD-L1 expression was upregulated. However, due to the presence of CuX-P in the TME, the subsequent replenishment of PD-L1 at the tumor surface again binds CuX-P for internalization. This feedback loop continuously blocks and depletes PD-L1 on the tumor surface and promotes the enrichment of CuX-P in the tumor thereby inducing cuproptosis, which stimulates a strong anti-tumor immune response in the mouse model after laser irradiation of CuX-P treatment. Shi et al. reported a novel copper chelator, JYFY-001, to inhibit the growth of CRC by chelating copper ions, and the combination of JYFY-001 with PD-1 inhibitors had a better inhibitory effect on CRC [231]. Moreover, Shen et al. prepared an injectable copper-induced hydrogel containing PD-L1 antibody and nitric oxide (NO) donor, which could significantly increase apoptosis of tumor cells, induce immune cell death, and be able to increase infiltration of cytotoxic T-lymphocytes (CTLs) by photothermal therapy, thereby reversing TME to improve the therapeutic efficacy of anti-PD-L1 in vivo [232].

#### CD47

CD47 is a widely expressed glycoprotein that binds to the SIRPα receptor to express a ‘don’t eat me’ signal thereby inhibiting phagocytosis by macrophages [233]. Zhan et al. constructed an engineered biomimetic nanoparticle enzyme (CD47@CCM-Lap-CuS NPs). By overexpressing CD47 protein on the BC cell membrane and binding it to SIRP-α on the surface of macrophages, the nanoparticle enzyme was able to evade phagocytosis by macrophages, prolong its circulation time in the bloodstream, and enrich in tumor tissues. This “don’t eat me” signaling function allows the nano-enzymes to target tumor cells more effectively and achieve synergistic effects of photothermal and chemodynamic therapy [234]. In addition, it was found that treatment of HCC cells with DSF/Cu promoted the activation and maturation of DCs. The combination of DSF/Cu and CD47 blockade further promoted the maturation of DCs and enhanced the cytotoxicity of CD8^+^ T cells, which could effectively activate the anti-tumor immune response and inhibit tumor growth [235].

#### CTLA-4

CTLA-4 is a classical immune checkpoint that negatively regulates T cell immune function at different stages of T cell activation [236]. One study developed DSF/MS-Cu-2 nanoparticles, and the combination treatment using DSF/MS-Cu-2 and anti-CTLA-4 antibody significantly inhibited tumor growth, and the inhibition of tumor growth was more pronounced in the combination treatment group compared to the group using DSF/MS-Cu-2 or anti-CTLA-4 antibody alone [237].

### Chimeric antigen receptor T cell (CAR-T) therapy

CAR-T therapy is the current hotspot of immune cell therapy and has revolutionized the immunotherapy of tumors due to its potent efficacy. CAR-T therapy has been very effective in treating certain blood disorders [238], but in contrast, CAR-T has not been effective in treating solid tumors [239]. Researchers used copper to enhance the effectiveness of CAR-T therapy against solid tumors [240]. Specifically, CAR-Ts were reprogrammed by exposing them to DSF and copper plus ionizing radiation (IR) in stressed target cancer cells. The reprogrammed CAR-T cells acquired early memory-like features, potent cytotoxicity, enhanced in vivo expansion, persistence, and reduced depletion. Simultaneously, DSF/Cu- and IR-treated tumors were also reprogrammed to reverse the immunosuppressive TME. Such reprogrammed CAR-T induced potent, long-lasting memory responses and curative effects on solid tumors in multiple xenograft mouse models.

### Tumor vaccine

Tumor vaccines are also a hotspot of current research [241]. The rapid development of nanobiotechnology has facilitated the development of therapeutic tumor vaccines. These vaccines have a powerful role in preventing tumor metastasis and recurrence by stimulating the host’s intrinsic immune response through tumor antigens and subsequently generating a cascade of adaptive responses against cancer, transforming “cold” tumors into “hot” tumors [242]. In the previous section, we mentioned a nanovaccine called Cu-THBQ/AX that uses copper to modulate macrophages to kill tumors [208]. Furthermore, a copper nanoparticle-based tumor vaccine is presented by Shen et al. [243]. This nanosystem incorporates integrated phenylboronic acid (PBA)-functionalized poly(amidoamine) dendrimers of generation 5 (G5), copper sulfide nanoparticles, and cyclic GMP-AMP (cGAMP), an immune adjuvant (for short, G5-PBA@CuS/cGAMP) to act as a photothermal-triggered nanovaccine. With its exceptional photothermal conversion efficiency and strong protein adsorption properties, this nanosystem holds promise for the photothermal therapy of primary melanoma tumors. It is designed to simultaneously absorb antigens from whole tumor cells, leading to the in vivo formation of a photothermal-triggered dendritic nanovaccine. This nanovaccine can induce an anti-tumor immune response and inhibit the growth of distant tumors. Additionally, when melanoma cells are treated with laser irradiation in vitro, they can form copper sulfide nanoparticle/antigen complexes, which further bind to cGAMP to create prefabricated nanovaccines. These prefabricated nanovaccines have effectively inhibited primary tumor growth and prevented tumorigenesis.

### Non-specific immune enhancer

The rapid development of modern tumor immunity has been a boon to cancer patients, but not all tumors respond to immunotherapy, and the use of adjuvants can be used to increase the immunogenicity of immunotherapy without causing serious adverse effects [244]. Nanoparticles as exogenous substances can activate the innate immune system through pattern recognition receptors (PRRs) and act as non-specific immune enhancers. Copper ions and Cu-NPs can act as immune enhancers and promote immune responses [245]. As we mentioned earlier, copper ions can affect the immune system through mechanisms such as modulation of immune cells, generation of reactive oxygen radicals, and regulation of metal ion homeostasis. Cu-NPs can enhance the immune response by enhancing antigen presentation and activating immune cells. In addition, Cu-NPs can synergize with immune checkpoint inhibitors and immunostimulants to enhance antitumor effects in tumors [246]. However, there are some limitations on the application of Cu-NPs as immune enhancers and further studies are needed to explore their potential and safety.

## Utilization of cuproptosis in other treatments (Table 5; Fig. 6)

### Copper ionophore

Here we briefly introduce several copper ionophores.

ES is an anticancer drug that targets mitochondrial metabolism [247]. Initially thought to be an oxidative stress inducing drug, ES has now been found to inhibit cancer by inducing cuproptosis.ES has been shown in some clinical trials to be sufficiently safe and effective in some cancers such as CRC [248], BC [249], OC [250], and melanoma [251]. Not all cancer cells are sensitive to ES, but cells with enhanced mitochondrial metabolism, such as CSC, drug-resistant cancer cells, and those with low glycolytic activity, are sensitive to ES [252]. Therefore, in clinical trials, one should select cancer types with high mitochondrial metabolism and try to combine ES with platinum drugs, protease inhibitors, molecularly targeted drugs, or glycolysis inhibitors.

DSF can also promote the accumulation of copper in cells, which can trigger cuproptosis [5]. Mixtures of DSF with copper show selective targeting of cancer cells, targeting them more readily than normal cells, and also targeting cancer stem cells [253]. It has been demonstrated that DSF or (DSF/Cu) has anti-tumor effects on a variety of cancers, such as GBM [254], BC [255], OC [256] and oral carcinoma [256]. The efficacy of DSF alone is weak, and in combination with Cu the efficacy is good but the cytotoxicity needs to be kept controllable, so considering the safety of using DSF, the balance between DSF, which is weak in efficacy, and DSF/Cu, which is strong in cytotoxicity, should be fully explored, so that DSF can be used clinically in the comprehensive treatment of tumors. Second, DSF/Cu modulates the microenvironment and induces immunogenic cell death (ICD). However, how to prevent the excessive release of cytokines and inflammatory responses during antitumor therapy requires further research. In addition, when DSF is used as an adjuvant therapy for cancer, its pharmacologic interactions need to be further investigated [257].

Moreover, Yang et al. explored the feasibility of curcumin as a copper ion carrier, which can control the metabolism of lipids, RNA, NADH, and NADPH and promote cuproptosis in CRC cells [258].

### Copper chelator

Copper chelators have several roles in the treatment of cancer [259]. Copper chelators can inhibit angiogenesis and impair tumor growth and metastasis [260]. Moreover, copper chelators can reduce the intracellular copper concentration, which is high in most cancer cells, so they can affect the growth and survival of cancer cells. Copper chelator JYFY-001 can effectively inhibit the proliferation of CRC cells and enhance the efficacy of anti-PD-1 therapy [231], while copper chelators Trientine can enhance the sensitivity of chemotherapeutic agents to tumor cells. They can affect the responsiveness of tumor cells to chemotherapeutic agents and enhance the efficacy of chemotherapy by modulating copper-related signaling pathways [261].

### Chemotherapy

Copper transport mechanisms have a great impact on the efficacy of chemotherapeutic drugs [259]. Inhibition of ATOX1 using DC_AC50 enhanced the effect of platinum-based drugs [35]. Meanwhile, elevated levels of GSH, DLAT, ATP7A, and ATP7B were correlated with increased resistance to chemotherapeutic drugs [54, 60, 61, 96], furthermore, CuET could bypass GSH interference and reverse cisplatin resistance through cuproptosis effects [149].

### Radiotherapy

Excess copper in tumor cells may lead to enhanced antioxidant capacity, thereby increasing the resistance of tumor cells to radiotherapy. Copper may reduce the killing effect of radiotherapy on tumor cells by enhancing DNA repair and antioxidant capacity. Recent studies have shown a correlation between cuproptosis and radiosensitivity [77]. Copper metabolism MURR1 domain 10 (COMMD10) was found to enhance ferroptosis and radiosensitivity. IR induces a decrease in COMMD10, which increases intracellular copper ion concentration, leading to HCC radiotherapy resistance [262]. Specifically, IR-induced low expression of COMMD10 inhibited the ubiquitination degradation of HIF1α while disrupting its binding to HIF1α, promoting HIF1α nuclear translocation as well as the transcription of ceruloplasmin and SLC7A11, which collectively inhibited the ferroptosis of HCC cells. In addition, elevated ceruloplasmin promoted HIF1α expression by decreasing Fe, forming a positive feedback loop. Moreover, the researchers designed an oxygen generator loaded with copper ions (^Cu/AP^H-M), which acts as an effective carrier of copper ions and is essential for enhancing oxygenation in the TME. ^Cu/AP^H-M-mediated cuproptosis and intensification of radiotherapy combined with immunotherapy could effectively inhibit tumor growth [263]. Li et al. designed and developed a novel “smart” multifunctional copper-based nanocomposite (RCL@Pd@CuZ) for enhancing the sensitivity of radiotherapy [264]. Specifically, RCL@Pd@CuZ enhances radiotherapy sensitivity through amelioration of hypoxia, promotion of cuproptosis, depletion of GSH, enhancement of oxidative stress, and enhancement of X-ray uptake. to enhance radiation sensitivity, thereby effectively amplifying with greater DC maturation and CD8^+^ T cell infiltration. This nanoplatform offers a promising therapeutic modality for promoting radioimmunotherapy in cuproptosis-associated cancers.

### Photothermal therapy

Copper and photothermal therapy are linked to the treatment of cancer [65]. By regulating the balance of copper ions, the killing effect of photothermal therapy on tumor cells can be enhanced, thus improving the therapeutic effect. In turn, photothermal therapy can increase the accumulation of copper ions in tumor cells, thereby enhancing the effects of cuproptosis [230, 232, 234, 243].

### Nano therapy (Table 4)

Copper has an important role in nano therapy [65]. Copper nanoparticles can be used in photothermal and chemo dynamic therapies, as well as to induce cuproptosis by releasing copper. In addition, these nanoparticles can enhance anti-tumor effects by modulating the TME and immunotherapy [152, 159, 178, 237, 243].

Copper-based nanomedicines induce cuproptosis in a variety of ways, causing tumor cell death while also activating a strong anti-tumor immune response via ICD.

Luo et al. proposed a copper (II)-based metal-organic framework nanoplatform (ES-Cu-MOF), which could effectively release Cu^2+^ and ES to induce cuproptosis in tumor cells. In addition, ES-Cu-MOF significantly triggered the ICD to activate the anti-tumor immune response. The ICD activated DCs, which led to an increase in the infiltration of cytotoxic CD8^+^T cells, which thereby enhancing the anti-tumor immune response and successfully inhibit the growth of fibrosarcoma [265]. Chang et al. designed a nanomedicine that efficiently assembled copper ions into polydopamine nanostructures (PDA-DTC/Cu) for reprogramming of copper metabolism in tumors, mediated by diethyldithiocarbamate (DTC) coordination [266]. Specifically, deposited Cu^2+^ induces tumor cell cuproptosis as well as disrupts copper metabolic homeostasis by disrupting mitochondrial function and limiting ATP energy supply, which catalyzes inhibition of ATP7A and ATP7B expression in tumor cells to enhance cuproptosis. Meanwhile, killed tumor cells induce ICD and stimulate immune response. In addition, PDA-DTC/Cu NPs could promote the repolarization of TAM, thereby alleviating the tumor immunosuppressive microenvironment. Lu et al. developed a self-amplifying cuproptosis nanoplatform (Cel-Cu NP) utilizing celastrol (Cel), a natural product isolated from medicinal plants. Cel-Cu NP, in addition to inducing cuproptosis, reduces GSH levels, thereby promoting cuproptosis. This self-amplifying cuproptosis further activates the ICD, which triggers a powerful immune response. Combined with ICB, Cel-Cu NP effectively eradicated metastatic tumors in a mouse lung metastasis model [267]. Wang et al. designed nanomedicines to potently kill triple-negative breast cancer (TNBC) cells through the synergistic effect of apoptosis and cuproptosis and the activation of the immune system [268]. They synthesized two precursors: PEG-TK-DOX, a ROS-responsive precursor, and PEG-DTPA-SS-CPT, a GSH-responsive precursor. These precursors were prepared by self-assembling and chelating the Cu^2+^ nanoparticles, PCD@Cu, loaded with both doxorubicin (DOX), camptothecin (CPT), and Cu^2+^. elevated levels of fros and GSH in TNBC cells disrupted the PCD@Cu structure, leading to the release of Cu^+^, DOX, and CPT, and the depletion of GSH. DOX and CPT triggered apoptosis accompanied by ICD in TNBC cells. Meanwhile, PCD@Cu down-regulated the ATP7B expression, leading to significant copper accumulation. This further induced cuproptosis, displaying potent anti-tumor ability. In addition, PCD@Cu also has a favorable biosafety profile.

High levels of lactic acid in TME not only promote tumor development and metastasis, but also induce immune escape and inhibit anti-tumor therapy [269]. Yu et al. reported an acoustically triggered cascade lactate depletion strategy for cancer cuproptosis immunotherapy using a semiconducting polymer nanoreactor (SPNLCu) [270]. The SPNLCu mainly contains semiconducting polymer as an acoustic sensitizer, lactate oxidase (LOx) coupled via ROS-cleavable linkers, and chelated Cu^2+^. Under ultrasound irradiation, LOx is released and catalyzes the consumption of lactate to produce hydrogen peroxide (H_2_O_2_). Cu^2+^ is reduced to Cu + in TME, which reacts with the generated H_2_O_2_ to produce hydroxyl radicals (ꞏOH), further facilitating the release of LOx. Thus, the sonar-triggered cascade release of LOx achieves an efficient depletion of lactate, thereby alleviating the immunosuppressive effects of lactate. In addition, toxic Cu^+^-induced cuproptosis can cause ICD, which activates the anti-tumor immune effect. Similarly, Xu et al. designed a nanomedicine targeting ccRCC to enhance chemodynamic therapy (CDT), activation of cuproptosis, and tumor immunotherapy through ccRCC cell membrane-modified CuO@Gd_2_O_3_ yolk-like particles (CGYL) loaded with LOx (mCGYL-LOx) [271]. Taking advantage of the homologous targeting of Renca cell membranes, mCGYS-LOx can efficiently release LOx and copper ions. LOx catalyzes excess lactic acid in Renca cells to H_2_O_2_, which is subsequently converted by copper ions to the highly toxic ꞏOH that promotes tumor CDT. at the same time, excess copper ions efficiently trigger tumor cuproptosis. these synergistic effects promote ICD leading to DC maturation and infiltration of immune effector cells. In addition, LOx-mediated lactate depletion downregulated PD-L1 expression and impaired immune escape from the tumor. In addition, mCGYL-LOx improved T_1_-weighted MRI signals for accurate diagnosis of ccRCC.

Hypoxic TME hindered the sensitivity of cuproptosis and inhibited the antitumor immune response. Huang et al. successfully immobilized and functionalized catalase (CAT) with long single-stranded DNA containing multivalent CpG sequences by the rolled-circle amplification (RCA) technique to obtain an enzyme-cored spherical nucleic acid nanoplatform (CAT-ecSNA-Cu) for delivery of copper ions for cuproptosis [272]. CAT enhances mitochondrial respiration by catalyzing the conversion of H_2_O_2_ to O_2_, thereby sensitizing cuproptosis. Simultaneously, increased tumor oxygenation inhibited HIF-1 expression and alleviated immunosuppressive TME. Notably, cuproptosis induced ICD, promoted DC maturation, and enhanced antigen presentation through polyCpG-supported toll-like receptor 9 (TLR9) activation. This strategy of enhancing cuproptosis-mediated antitumor immune responses by alleviating hypoxia effectively promotes the activation and proliferation of effector T cells, ultimately leading to long-term immunity against cancer.

Phototherapy likewise induces copper-based nanomedicines to trigger cuproptosis and ICD. Zafar et al. developed bacterial membrane-coated copper-human serum albumin nanocomplexes loaded with gold nanocages (BAu-CuNCs) [273]. Under near-infrared laser irradiation, copper-human serum albumin (Cu-HSA) release was enhanced and induced cuproptosis by releasing Cu^2+^ via disulfide exchange reaction with intratumoral GSH. Subsequently, the cuproptosis effect triggered the ICD, which achieved anti-tumor immunity through the massive production of CD8^+^ T cells and CD4^+^ T cells. Meanwhile, gold nanocages (AuNCs) promoted the overproduction of ROS, inhibited tumor glycolysis as well as reduced lactate and ATP. While the combined effects of low lactate levels, ATP reduction and GSH depletion further enhanced cuproptosis sensitivity. In addition, low lactate secretion inhibits Tregs, thereby enhancing anti-tumor immunity.

Similarly, Liang et al. proposed a novel approach using copper-coordinated nano-assemblies (CCNAs), which were created by combining the photosensitizer Zinc Phthalocyanine (ZnPc)-chemotherapeutic (DOX) prodrug with the thioketa (TK) spacer and the IDO inhibitor (1-methyltryptophan, 1-MT) as a cornerstone for Cu^2+^-coordinated self-assembly for combined apoptosis-cuproptosis and immunotherapy [274]. Under near-infrared laser irradiation, the ZnPc fraction of CCNAs exhibited a photodynamic effect to generate ROS. this triggered the release of DOX, leading to enhanced apoptosis of tumor cells. In addition, the presence of Cu^2+^ in CCNAs not only enhanced the photodynamic process by catalyzing oxygen generation, but also promoted cuproptosis, followed by the cuproptosis effect that triggered the ICD response. Meanwhile, released 1-MT complemented this response by reversing the immunosuppressive TME, synergistically enhancing antitumor immunity, and inhibiting the growth of primary and distant tumors.

Zu et al. developed a metabolically targeted Cu_2−x_S (MACuS) nano-agent. MACuS nano-agents can specifically target tumors via glucose transporter receptor 1, and near-infrared laser-II irradiation not only directly thermally ablates tumor cells, but also promotes highly efficient cuproptosis and enhances ROS-induced tumor cytotoxicity. The triple action of MACuS induced ICD, which triggered a systemic anti-tumor immune response and showed effective inhibition of growth, metastasis and recurrence in mouse and rabbit breast cancer models [275].

Jiang et al. proposed a novel copper-based nanomaterials (CHP) consisting of a phosphate skeleton doped with copper and hafnium ions (Hf^4+^) and then modified with polyvinylpyrrolidone (PVP) [276]. CHP can achieve tumor radio sensitization at low X-ray dose by depleting tumor endogenous GSH, alleviating tumor hypoxemia and repolarization of M2-phenotype macrophages, and reprogramming TME, and gradually accumulate intra-tumor ROS and enhance cuproptosis. In addition, cuproptosis can amplify radiotherapy induced anti-tumor immunity by activating ICD, ultimately producing a strong anti-tumor immune response and long-term immunity. The study sheds new light on the treatment of BC.

Combined with the above research results, copper-based nanomedicines induce cuproptosis by releasing copper ions through photodynamic therapy, chemodynamic therapy, etc., and also activate the ICD to cause a strong anti-tumor immune response, so cuproptosis may has an extremely close relationship with the ICD, which provides a powerful new idea for anti-tumor therapy.

## Challenges and Perspectives

In hypoxic TME, due to the Warburg effect, tumor cells are more inclined to generate energy through glycolysis, often showing high glycolysis levels and low oxidative phosphorylation [82]. Whereas copper induces mitochondrial proteotoxic stress ultimately leading to cuproptosis mainly by affecting the TCA cycle, this mechanism tends to result in inhibition of cuproptosis. In addition, due to the low level of intracellular copper ions, copper ionophores are often needed to transport exogenous copper into the cell to induce cuproptosis. Clinical trials of copper ionophores such as ES and DSF are underway. Given the heterogeneous nature of the tumors, copper metabolism varies in different patients and tumors, so it is necessary to combine different tumors with other drugs such as chemotherapeutic drugs and immunotherapeutic drugs in combination therapy, and considering safety of the drugs used is also required.

In animal models, both the dose and the duration of copper intake are related to the accumulation of copper in the liver, oxidative stress and the degree of liver injury, so when using copper-containing drugs, the dose of the drugs should be strictly controlled to avoid excessive intake of copper. In addition, it is important to consider individual differences in patients and adjust the drug dose according to their weight, age, health status and other factors to minimize the risk of copper toxicity. Regularly monitor the patient’s copper levels and liver function to assess copper accumulation and potential toxicity, and promptly detect and manage signs of copper toxicity. Since copper toxicity is associated with oxidative stress, it can be reduced by antioxidant therapy. Finally, drug design to develop drugs that can specifically target diseased cells and minimize the impact on normal cells [67].

With the vigorous development of nanomaterials, it is hoped that more copper-based nanomedicines will be available in the future to treat tumors through the unique mechanism of cuproptosis induction.

## Conclusion

This review reveals the important roles of copper metabolism and cuproptosis in cancer development and highlights their regulatory roles in TME and immune response. The potential of CRGs as prognostic indicators and therapeutic targets offers new strategies for cancer treatment. Future studies should further explore the use of copper metabolism modulators and cuproptosis inducers in clinical therapy and how they can synergize with other therapeutic approaches to improve the efficiency and efficacy of cancer treatment.

## Data Availability

No datasets were generated or analysed during the current study.

## Abbreviations

CRGs: cuproptosis-related genes
TME: tumor microenvironment
ATP7A/B: ATPase copper transporting alpha/beta
STEAP: six-transmembrane epithelial antigen of prostate
DCYTB: duodenal cytochrome b
SLC31A1/2: solute carrier family 31 member 1/2
CTR1/2: copper transporter 1/2
ZNF711: zinc finger protein 711
SP1: specificity protein 1
PTBP1: polypyrimidine tract binding protein 1
AMPK: AMP-activated protein kinase
ROS: reactive oxygen species
DMT1: divalent metal transporter protein 1
ZnT1: zinc transporter protein 1
EC: endometrial cancer
ACC: adrenocortical carcinoma
MESO: mesothelioma
CCS: copper chaperone protein for superoxide dismutase
ATOX1: antioxidant 1 copper chaperone protein
COX17: cytochrome c oxidase copper chaperone protein 17
SOD1: superoxide dismutase 1
ATP: adenosine triphosphate
NSCLC: non-small cell lung cancer
MEK1/2: mitogen-activated extracellular signal-regulated kinase 1/2
MEMO1: mediator of ErbB2-driven cell motility 1
LOX: lysyl oxidase
HIF1: hypoxia-inducible factor 1
SCO1: synthesis of cytochrome c oxidase 1
COA6: cytochrome c oxidase assembly factor 6
MT-CO1/COX1: mitochondria-encoded cytochrome c oxidase 1
MT-CO2/COX2: mitochondria-encoded cytochrome c oxidase 2
COX11: cytochrome c oxidase copper chaperonin 11
CYCS: cytochrome c somatic cell
LUAD: lung adenocarcinoma
MT: metallothionein
GSH: glutathione
OC: ovarian cancer
ES: elesclomol
DSF: disulfiram
BSO: buthionine sulfoximine
FDX1: ferredoxin 1
LIAS: lipoic acid synthetase
DLAT: dihydrolipoamide S-acetyltransferase
TCA: mitochondrial tricarboxylic acid
COAD: colon adenocarcinoma
DLBC: diffuse large B-cell lymphoma
GBM: glioblastoma multiforme
OV: ovarian serous cystadenocarcinoma
PAAD: pancreatic adenocarcinoma
PRAD: prostate adenocarcinoma
READ: rectal adenocarcinoma
SKCM: skin cutaneous melanoma
STAD: stomach adenocarcinoma
THYM: thymoma
UCEC: uterine corpus endometrial carcinoma
OS: osteosarcoma
UCS: uterine carcinosarcoma
AML: acute myeloid leukemia
BRCA: breast invasive carcinoma
CCA: cholangiocarcinoma
HNSCC: head and neck squamous cell carcinoma
KICH: kidney chromophobe
ccRCC: clear cell renal cell carcinoma
KIRP: kidney renal papillary cell carcinoma
LUSC: lung squamous cell carcinoma
PCPG: pheochromocytoma and paraganglioma
TGCT: testicular germ cell tumor
THCA: thyroid cancer
AKGDH: alpha-ketoglutarate dehydrogenase
HCC: hepatocellular carcinoma
PD-L1: programmed cell death ligand 1
PDHA1: pyruvate dehydrogenase E1 component subunit alpha
acetyl-CoA: acetyl-coenzyme A
SAKI: sepsis-induced acute kidney injury
CSCC: cervical squamous cell carcinoma
CESC: cervical squamous cell carcinoma and endocervical adenocarcinoma
PCA: prostate cancer
NADH/NAD^+^: nicotinamide adenine dinucleotide
PDHB: pyruvate dehydrogenase E1 subunit beta
PDH: pyruvate dehydrogenase
BLCA: bladder cancer
ESCA: esophageal cancer
CDKN2A: cyclin-dependent kinase inhibitor 2 A
GLS: glutaminase
BC: breast cancer
PDAC: pancreatic ductal adenocarcinoma
MTF1: metal-regulatory transcription factor 1
EMT: epithelial to mesenchymal transition
GC: gastric cancer
PD-1: programmed cell death protein 1
CTLA-4: cytotoxic T lymphocyte antigen 4
MELK: maternal embryonic leucine zipper kinase
lncRNAs: long noncoding RNAs
SERPINE1: serine protease inhibitor clade E member 1
NK cells: natural killer cells
CRC: colorectal cancer
4-OI: 4-octyl itaconate
GAPDH: glyceraldehyde 3-phosphate dehydrogenase
Nrf2: nuclear factor erythroid 2-related factor 2
ADM: adrenomedullin
FOXO3: forkhead box O3
CP: coordination polymer
PDE3B: phosphodiesterase 3B
KRT6B: keratin 6B
TFP-Cu: Cu ion-coordinated Tremella fuciformis polysaccharide
GCSH: glycine cleavage system protein H
CRLs: cuproptosis-related lncRNAs
TIME: tumor immune microenvironment
WASF2: WASP family member 2
CuAS: cuproptosis activation scoring
VEGF: vascular endothelial growth factor
HFn: H-ferritin
BBB: blood-brain barrier
TfR1: transferrin receptor 1
CSC: cancer stem cells
HBO: hyperbaric oxygen
CuET@PH: NPs polydopamine and hydroxyethyl starch stabilized copper-diethyldithiocarbamate nanoparticles
IL-2: interleukin-2
CuO: NPs copper oxide nanoparticles
Tregs: regulatory T cells
TAMs: tumor-associated macrophages
LPS: lipopolysaccharide
CTMM: CuTCPP@MOF nanodots@Mannosamine
DAMPs: damage-associated molecular patterns
Lip@Fe-Cu-MOFs: thermosensitive liposomal formulation of bimetallic Fe-Cu metal-organic frameworks
MDSCs: myeloid-derived suppressor cells
IMCs: immature myeloid cells
G-MDSCs: granulocyte-like MDSCs
PMN-MDSCs: polymorphonuclear-like MDSCs
M-MDSCs: mononuclear phagocyte-like MDSCs
CuNG: copper N-(2-hydroxy acetophenone) glycinate
DCs: dendritic cells
CAR-T: chimeric antigen receptor T cell
IR: ionizing radiation
G5-PBA@CuS/cGAMP: phenylboronic acid (PBA)-functionalized poly(amidoamine) dendrimers of generation 5 (G5), copper sulfide nanoparticles, and cyclic GMP-AMP (cGAMP)
PRRs: pattern recognition receptors
Cu-NPs: copper nanoparticles
COMMD10: copper metabolism MURR1 domain 10
ICD: immunogenic cell death
ES-Cu-MOF: copper (II)-based metal-organic framework nanoplatform
DTC: diethyldithiocarbamate
Cel: celastrol
TNBC: triple-negative breast cancer
CGYL: CuO@Gd2O3 yolk-like particles
mCGYL-LOx: CuO@Gd2O3 yolk-like particles (CGYL) loaded with LOx
CAT: catalase
RCA: rolled-circle amplification
TLR9: toll-like receptor 9
BAu-CuNCs: bacterial membrane-coated copper-human serum albumin nanocomplexes loaded with gold nanocages
Cu-HAS: copper-human serum albumin
AuNCs: gold nanocages
CCNAs: copper-coordinated nano-assemblies
ZnPc: Zinc Phthalocyanine
TK: thioketa
1-MT: 1-methyltryptophan, 1-MT
Hf4+: hafnium ions
PVP: polyvinylpyrrolidone
